# Antibody recognition of the Pneumovirus fusion protein trimer interface

**DOI:** 10.1371/journal.ppat.1008942

**Published:** 2020-10-09

**Authors:** Jiachen Huang, Darren Diaz, Jarrod J. Mousa

**Affiliations:** 1 Department of Infectious Diseases, College of Veterinary Medicine, University of Georgia, Athens, GA, United States of America; 2 Center for Vaccines and Immunology, College of Veterinary Medicine, University of Georgia, Athens, GA, United States of America; University of Texas Medical Branch / Galveston National Laboratory, UNITED STATES

## Abstract

Human metapneumovirus (hMPV) is a leading cause of viral respiratory infection in children, and can cause severe lower respiratory tract infection in infants, the elderly, and immunocompromised patients. However, there remain no licensed vaccines or specific treatments for hMPV infection. Although the hMPV fusion (F) protein is the sole target of neutralizing antibodies, the immunological properties of hMPV F remain poorly understood. To further define the humoral immune response to the hMPV F protein, we isolated two new human monoclonal antibodies (mAbs), MPV458 and MPV465. Both mAbs are neutralizing *in vitro* and were determined to target a unique antigenic site using competitive biolayer interferometry. We determined both MPV458 and MPV465 have higher affinity for monomeric hMPV F than trimeric hMPV F. MPV458 was co-crystallized with hMPV F, and the mAb primarily interacts with an alpha helix on the F2 region of the hMPV F protein. Surprisingly, the major epitope for MPV458 lies within the trimeric interface of the hMPV F protein, suggesting significant breathing of the hMPV F protein must occur for host immune recognition of the novel epitope. In addition, significant glycan interactions were observed with a somatically mutated light chain framework residue. The data presented identifies a novel epitope on the hMPV F protein for epitope-based vaccine design, and illustrates a new mechanism for human antibody neutralization of viral glycoproteins.

## Author summary

Human metapneumovirus (hMPV) is a common cause of lower respiratory tract infection in children, the elderly, and the immunocompromised. There is currently no approved vaccine or therapeutic to prevent or treat hMPV infection. The hMPV fusion (F) protein is the sole target of antibodies that neutralize hMPV. Here we describe new human mAbs that bind a unique epitope on the hMPV F protein. The mAbs were mapped to a new antigenic site on the hMPV F protein located within the trimeric interface of the hMPV F protein based on competitive biolayer interferometry and X-ray crystallographic experiments. This is the first example of a human mAb that binds inside the trimeric interface of a viral glycoprotein and neutralizes the virus. The results suggest such antibodies may exist for other viral fusion proteins, and identify a new epitope on the hMPV F protein for epitope-based vaccine design.

## Introduction

Human metapneumovirus (hMPV) is a leading cause of viral respiratory infections in children, the majority of whom are seropositive for hMPV by five years of age [1]. Although hMPV was discovered in 2001 [2], there are no vaccines or therapeutics approved to prevent or treat viral infection. Similar to other respiratory pathogens, children, the elderly, and the immunocompromised are the major groups for which hMPV infection may require hospitalization [3–11]. Several reports have demonstrated hMPV infection can be lethal in both adults and children. In particular, haemopoietic stem cell transplant patients are at high risk of severe hMPV infection [10–13], and several outbreaks of hMPV in nursing homes have been reported [14–16]. In addition, fatal hMPV has been observed in one child during an outbreak of hMPV in a daycare center [17]. hMPV is also a significant cause of febrile respiratory illness in HIV-infected patients [18], and has been linked to exacerbations of chronic obstructive pulmonary disease [19]. Co-circulation of hMPV was observed during the SARS outbreak of 2003 [20–22], and similar observations have been made during the current SARS-CoV-2 pandemic [23,24], suggesting hMPV interacts with other circulating respiratory viruses.

hMPV circulates as two genotypes, A and B, and based on the sequence variability of the surface proteins, hMPV is further grouped into four subgroups, A1, A2, B1, and B2 [25,26], and two additional subgroups, A2a and A2b, have been proposed [12]. hMPV has three surface glycoproteins, the small hydrophobic (SH), the attachment (G), and the fusion (F) proteins. The hMPV SH protein has been demonstrated to have viroporin activity [27], while the hMPV G protein has been hypothesized to be involved in cellular attachment [28]. The hMPV F protein is indispensable for hMPV infection, and is highly conserved among hMPV subgroups [29]. Furthermore, the hMPV F protein is the sole target of neutralizing antibodies [30]. Although the hMPV G protein is thought to interact with proteoglycans, the hMPV F protein can interact with glycans in the absence of hMPV G [31]. The hMPV F protein contains a highly conserved RGD motif that has been proposed as a key region in receptor binding to cellular integrins [32,33]. The entry of hMPV into the host cell can occur by cell membrane or endosomal membrane fusion [34].

Both hMPV and the related respiratory syncytial virus (RSV) are members of the *Pneumoviridae* family, and share a structurally similar F protein that has approximately 30% homology between the two viruses. For both viruses, the F protein has two long-lived conformations, the pre-fusion and post-fusion states [35]. Both RSV and hMPV lacking the G protein can infect cells *in vitro*, although these viruses are attenuated *in vivo* [36]. Both RSV G and hMPV G proteins are immunogenic, yet only antibodies to RSV G are neutralizing [30,37]. The pre-fusion conformation of the Pneumovirus F protein is meta-stable, and stabilized versions of both hMPV F [38] and RSV F [39,40] have been generated. The RSV F protein was initially stabilized in the pre-fusion conformation using cysteine substitutions to lock the protein in the pre-fusion state by disulfide bonds, and through cavity-filling mutations to prevent transition to the post-fusion state. This Ds-Cav1 construct has been further developed for clinical trials, and has shown promise in a phase I clinical trial [41]. Additional constructs for RSV F have focused on stabilizing the α4–α5 loop through proline mutations [40]. A similar approach was undertaken for the hMPV F protein, whereby a A185P mutation was introduced to stabilize the pre-fusion conformation [38].

The hMPV F protein contains a single site that is cleaved to convert the polypeptide F_0_ protein into the meta-stable disulfide-linked F_1_-F_2_ pre-fusion homotrimer. This is in contrast to RSV F, which contains two furin cleavage sites flanking the p27 peptide fragment. The cleavage enzyme for hMPV F *in vivo* is currently unknown, although cleavage can be accomplished by trypsin *in vitro* [42]. Post-fusion hMPV F has been generated by removing the fusion peptide and incorporating one furin cleavage site from RSV F [43]. X-ray crystal structures of the hMPV F protein from the A1 subgroup have been determined in the pre-fusion and post-fusion conformations [38,43]. Both proteins were expressed in CV-1 cells using a vaccinia virus expression system, although stabilized versions for routine HEK293F or CHO cell line expression have not yet been generated.

For RSV F, the pre-fusion conformation contains antigenic sites Ø [44] and V [45] located on the head of the RSV F protein, which elicit a more potent neutralizing antibody response as compared to the post-fusion conformation [44,45]. Furthermore, the human antibody response to RSV infection is primarily focused on these pre-fusion-specific epitopes [46]. For hMPV F, data using human serum has shown that the preponderance of hMPV F-specific human antibodies bind both pre-fusion and post-fusion F conformations, which has been proposed is due to differential glycan positioning on the head of the hMPV F protein as compared to the RSV F protein [38]. Although several monoclonal antibodies (mAbs) have previously been isolated that recognize the hMPV F protein [43,47–54], the predominant antigenic sites targeted by the human antibody response are unclear. A panel of rodent-derived mAbs was initially used to map the neutralizing epitopes on the hMPV F protein using viral escape mutants [47,48]. The known antigenic sites on the hMPV F protein include antigenic sites III, IV, and an unnamed site targeted by mAb DS7 [35]. DS7 was isolated from a human phage display library [49], and was co-crystallized with a fragment of the pre-fusion hMPV F protein [55]. Several mAbs isolated have been found to cross-neutralize RSV and hMPV, including MPE8 [51] and 25P13 [52] (site III), and 101F [43], 54G10 [50], and 17E10 [53] (site IV). In addition, we have recently isolated a panel of human mAbs targeting site III and the DS7 epitope [54]. One of these mAbs, MPV364, competes for binding at antigenic site III, but does not cross-react with RSV F, suggesting closer examination of hMPV F epitopes is required to improve knowledge on the optimal epitopes for elicitation of neutralizing antibodies. In this study, we isolated new human mAbs to further identify the epitopes on the hMPV F protein recognized by the human immune system.

## Results

### Isolation of human antibodies to the hMPV F protein

In our efforts to identify the major antigenic epitopes on the hMPV F protein, we isolated mAbs from human subjects using hybridoma technology [56]. As hMPV infection and exposure is not routinely tested in patients, and the majority of individuals are seropositive for hMPV infection [57], we isolated mAbs from two healthy human subjects. Two new mAbs were isolated against the recombinantly expressed hMPV B2 F protein (**S1 Table**) expressed in HEK293F cells [54]. MPV458 and MPV465 were isolated from two different donors, and have isotypes of IgG_3_ and kappa, and IgG_1_ and lambda, respectively. MPV458 utilizes V_H_3-30, J_H_3, D_H_2, V_K_1-33, and J_K_5, while MPV465 utilizes V_H_3-33, J_H_5, D_H_3-22, V_L_-47, and J_L_3. The heavy chain complementarity determining region (HCDR) 3 loop length differs dramatically between the two mAbs as the HCDR3 loop for MPV458 is just 8 amino acids, while MPV465 has a 21 amino acid long CDR3 loop (**S2 Table**).

### Epitope identification

**Fig 1:**
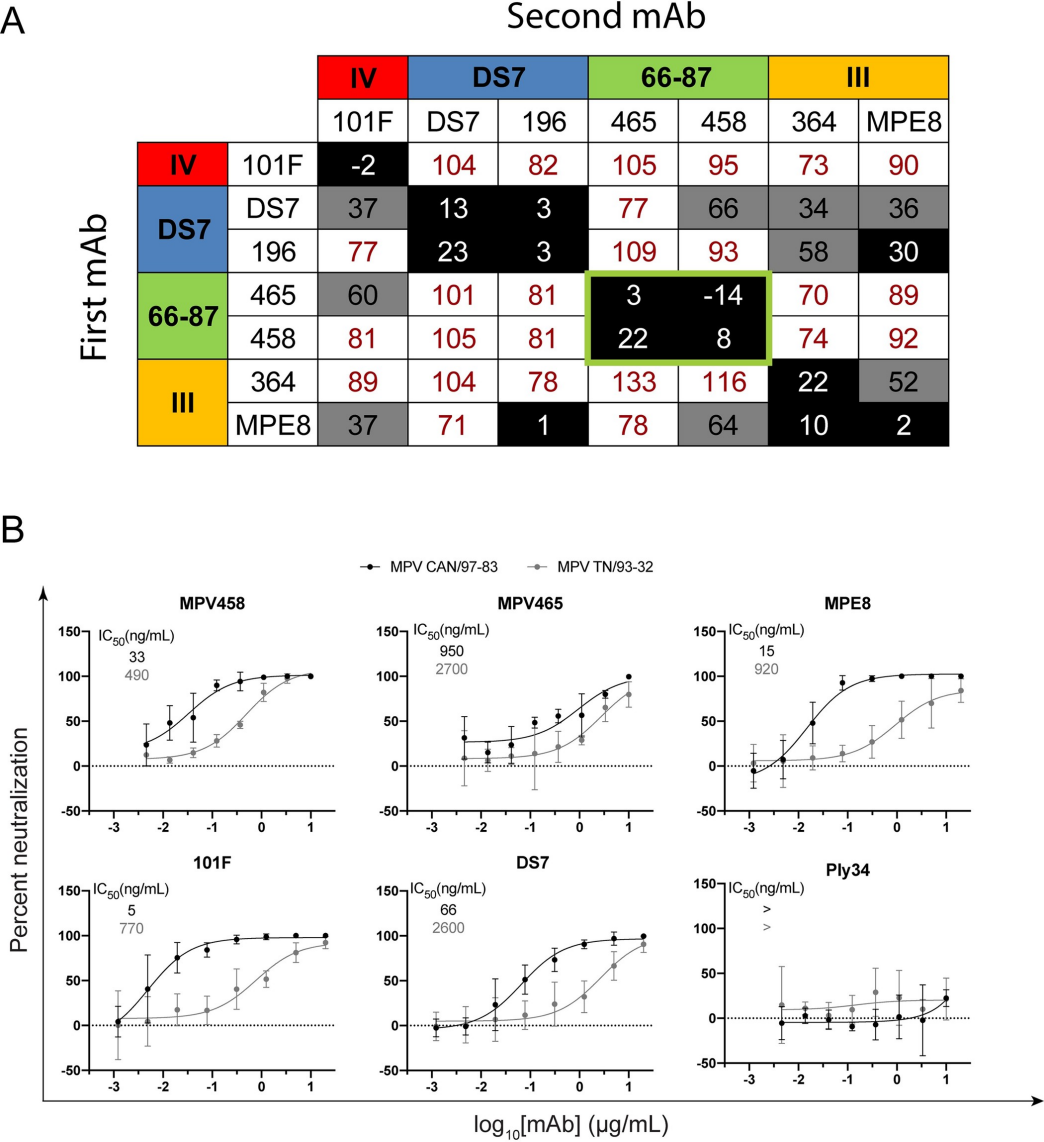
Epitope specificity and neutralizing properties of MPV458 and MPV465. (A) Epitope mapping of the hMPV F-specific mAb panel. Epitope control mAbs include 101F (site IV), DS7 and MPV196 (DS7 epitope), and MPV364 and MPE8 (site III). MPV465 and MPV458 do not compete with known mAbs, and compete with each other for binding, suggesting both mAbs bind at a previously undiscovered antigenic site. Data indicate the percent binding of the competing antibody in the presence of the primary antibody, compared with the competing antibody alone. Cells filled in black indicate full competition, in which ≤33% of the uncompeted signal was observed; cells in gray indicate intermediate competition, in which the signal was between 33% and 66%; and cells in white indicate noncompetition, where the signal was ≥66%. Antigenic sites are highlighted at the top and side based on competition binding with the control mAbs. (B) Plaque neutralization curves for MPV458 and MPV465 with controls. Both MPV458 and MPV465 are neutralizing, while MPV458 has neutralizing properties similar to MPE8 and 101F. IC_50_ values are inlaid in each curve. The pneumococcal-specific antibody Ply34 was used as a negative control. Data points are the average of three replicates and error bars are 95% confidence intervals. Data are shown from one experiment and are representative of two independent experiments. A mAb was considered neutralizing if >50% plaque reduction was observed at the highest concentration.

To identify the antigenic epitopes targeted by the isolated mAbs, we performed epitope binning using competitive biolayer interferometry [58]. Previously discovered mAbs with known antigenic epitopes were utilized as mapping controls, including mAbs 101F [59] (site IV), MPV196 [54] and DS7 [49] (DS7 epitope), and MPE8 [51] and MPV364 [54] (site III) (**Fig 1A; S1 Fig**). These previously discovered mAbs bind both pre-fusion and post-fusion conformations of the hMPV F protein with similar affinity, except for MPE8, which has higher affinity for the pre-fusion conformation of hMPV F. Anti-penta-HIS biosensors were loaded with the hMPV 130-BV F [38] protein (A1 subgroup, **S1 Table**) and then loaded with one hMPV F-specific mAb, followed by exposure to a second mAb. mAbs MPV458 and MPV465 did not compete with any of the mapping control mAbs, yet competed for binding with each other, suggesting these two mAbs bind to a unique antigenic site on the hMPV F protein.

### Neutralization and binding properties

**Fig 2:**
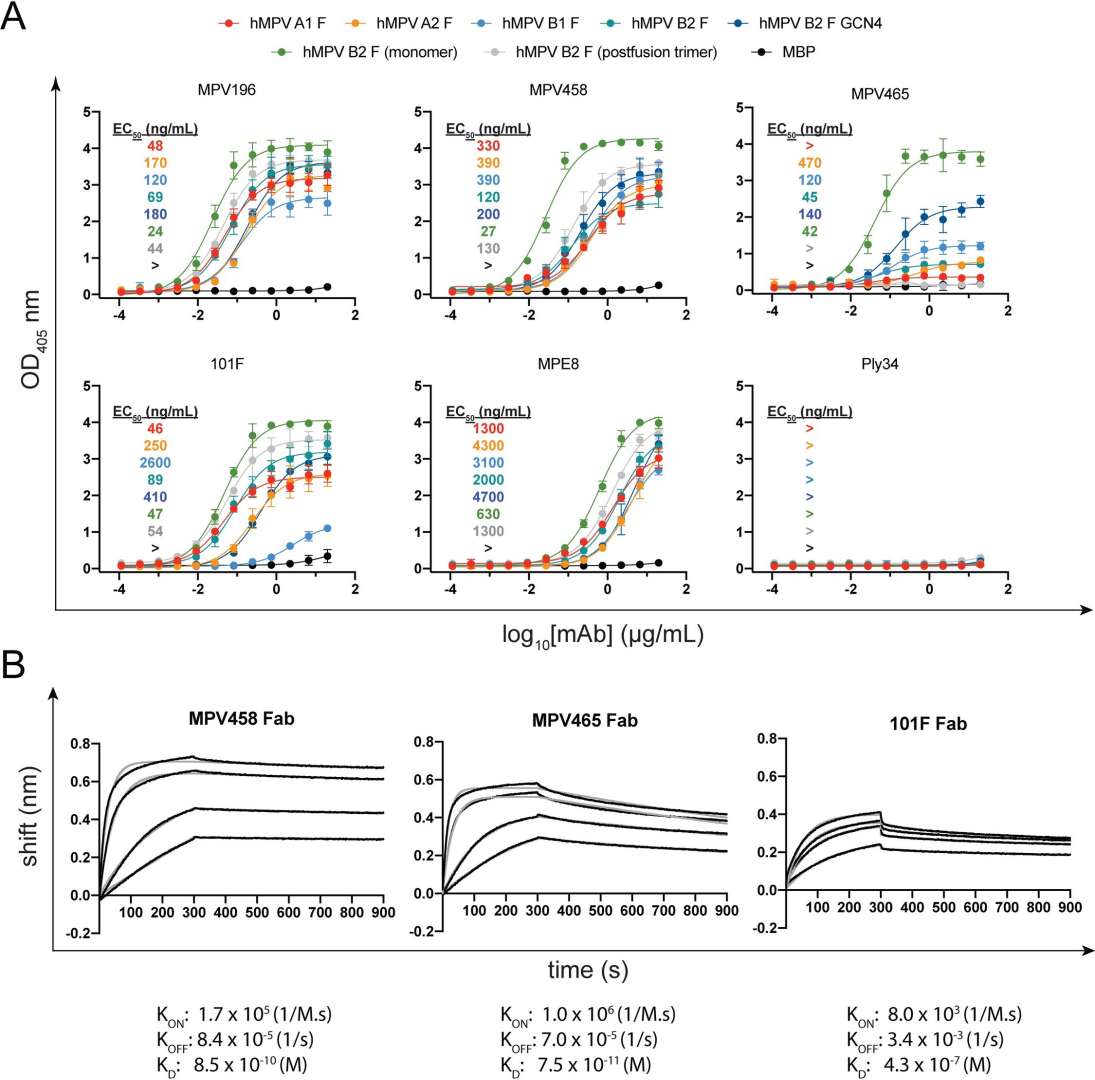
Binding measurements for MPV458, MPV465, and control mAbs. (A) ELISA binding curves for hMPV F-specific mAbs against a panel of hMPV F proteins including purified monomeric and trimeric hMPV B2 F protein that was treated with trypsin. Binding curves and EC_50_ values are colored according to the legend. MPV458 and MPV465 have lower EC_50_ values (higher affinity) for monomeric hMPV B2 F than trimeric hMPV B2 F. MPV458 and MPV465 bind to hMPV F proteins from all four subgroups. Data points are the average of four replicates and error bars are 95% confidence intervals. > indicates the calculated EC_50_ was either not in the range of the curve (due to an overall low signal) or a signal above 0.5 absorbance units was not detected at the highest concentration of 20 μg/mL. Each data point is the average of four replicates and error bars represent 95% confidence intervals. Data are representative of one experiment from two independent experiments. (B) Binding curves from biolayer interferometry. Biotinylated monomeric hMPV B2 F protein coated streptavidin biosensors were exposed to Fabs (MPV458 and MPV465: 10/5/1/0.5 μg/mL, 101F: 100/75/50/25 μg/mL) for 300 s before dissociating in buffer for 600 s. Binding constants are displayed underneath each graph and were averaged from the four replicates.

Plaque neutralization assays were performed to determine the neutralization properties of MPV458 and MPV465 against hMPV subgroup B2 (strain TN/93-32) and hMPV subgroup A2 (strain CAN/97-83) *in vitro* (**Fig 1B**). MPV458 neutralized hMPV with 50% inhibitory concentration (IC_50_) values of 33 ng/mL for MPV CAN/97-83 and 490 ng/mL for MPV TN/93-32, while MPV465 had IC_50_ values of 950 and 2700 ng/mL, respectively. The neutralization potency of MPV458 was comparable to mAbs MPE8 and 101F. We next determined the binding properties of MPV458 and MPV465 by ELISA and biolayer interferometry. For ELISA, the half-maximal effective concentration (EC_50_) values were used to quantify binding between mAbs across multiple hMPV F protein constructs (**Fig 2A; S2 Fig; S3 Fig; S1 Table**). Generating trimeric hMPV F can be achieved by treating purified protein with trypsin as previously described [43,54], although this process generates batch to batch variation of both pre-fusion and post-fusion conformations [54]. MPV458 binds to hMPV F proteins from all four hMPV F subgroups, while MPV465 showed binding to hMPV F proteins from subgroups A2, B1, and B2 (**Fig 2A**). We next assessed binding to exclusively monomeric (presumed pre-fusion) and trimeric (post-fusion) hMPV B2 F proteins that were treated with trypsin to induce cleavage (**Fig 2A; S3 Fig**), and MPV458 and MPV465 had higher binding to monomeric hMPV F than to trimeric hMPV F. MPV458 had a nearly five-fold lower EC_50_ to monomeric hMPV B2 F than to trimeric hMPV B2 F. MPV465 bound well to the hMPV B2 F monomer, while binding was completely abrogated binding to the post-fusion hMPV B2 F trimer. These data indicate the epitope for MPV458 and MPV465 is predominantly exposed on pre-fusion and/or monomeric hMPV F. Binding affinity of the mAbs was assessed by biolayer interferometry using monomeric hMPV B2 F protein (**Fig 2B**). Affinity measurements were completed by cleaving mAbs to Fab fragments and coupling biotinylated hMPV B2 F monomer to streptavidin biosensors. Association (K_ON_), dissociation (K_OFF_), and K_D_ values were comparable for MPV458 and MPV465 Fabs, and the Fabs had a 2–3 log higher K_D_ than the control mAb 101F. Binding K_D_ values were 0.85 nM, 0.075 nM, and 430 nM for Fabs of MPV458, MPV465, and 101F to monomeric hMPV B2 F protein, respectively.

### X-ray crystal structure of the hMPV B2 F + MPV458 complex

**Fig 3:**
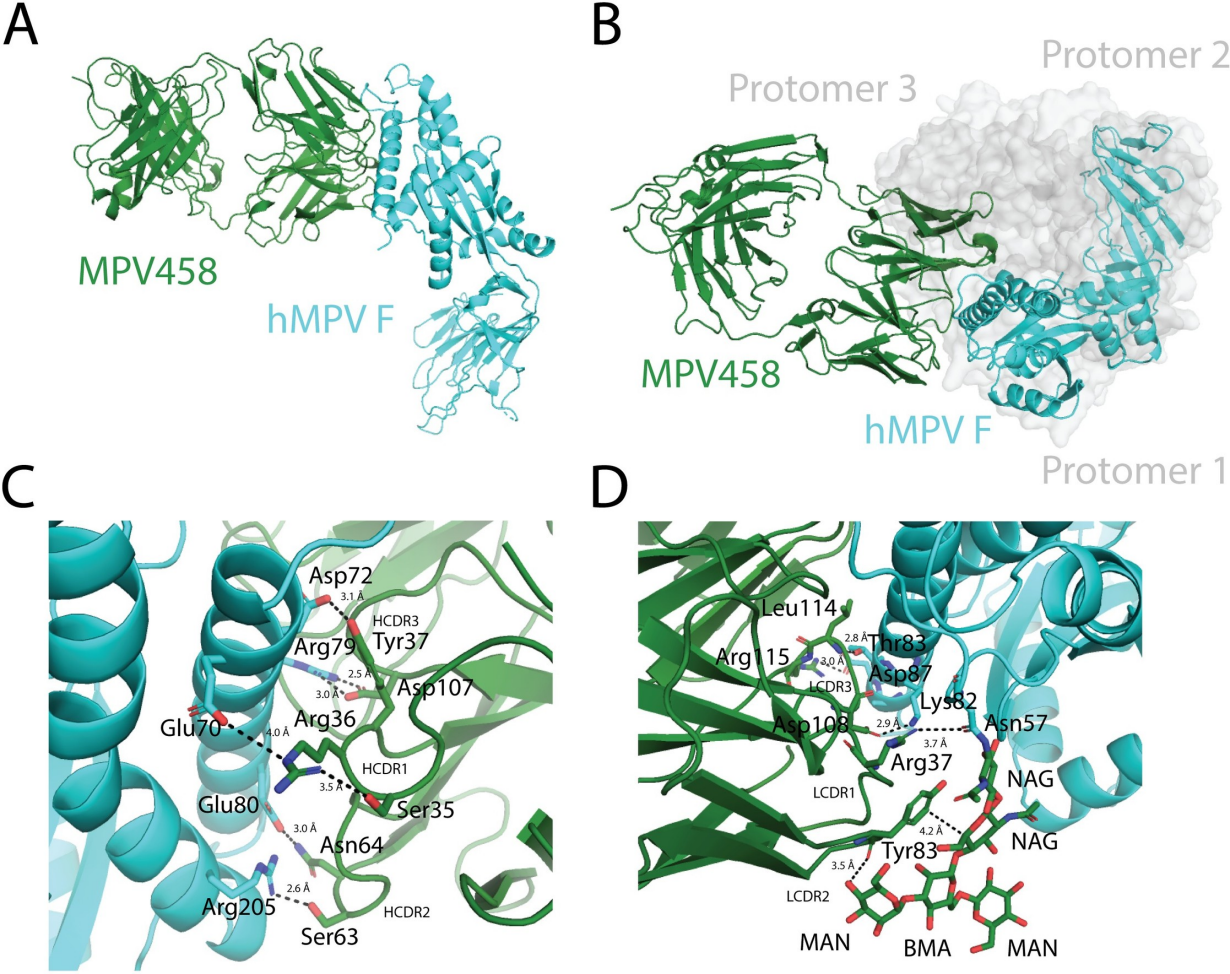
X-ray crystal structure of the hMPV B2 F + MPV458 Fab complex. (A) The asymmetric unit of the complex is displayed. Monomeric hMPV B2 F co-crystallized with one Fab of MPV458. (B) Overlay of the hMPV B2 F + MPV458 Fab complex with the previously determined X-ray crystal structure of hMPV A1 F in the pre-fusion conformation (MPV 115-BV, PDB: 5WB0). The hMPV F protein from each structure were overlaid in PyMol. MPV458 clashes with the trimeric structure. (C) Hydrogen bonding events observed between hMPV B2 F and the MPV458 Fab heavy chain. (D) Hydrogen bonding events observed between hMPV B2 F and the MPV458 light chain. The MPV458 light chain also interacts with an extended glycan patch linked from Asn57. CDR is complementarity determining region, FR is framework region. MPV458 numbering is in IMGT format.

To fully define the epitope targeted by the newly isolated mAbs, we co-crystallized the Fab of MPV458 in complex with hMPV B2 F. Trypsinization of hMPV B2 F generated trimeric and monomeric versions of hMPV F as assessed by size exclusion chromatography (**S4 Fig**). Cleavage of MPV458 and MPV465 mAbs to Fab fragments and subsequent addition of these Fabs to trypsinized trimeric hMPV B2 F resulted in monomeric hMPV F-Fab complexes (**S4 Fig; S5 Fig**). Although the hMPV B2 F trimer appeared to fall apart upon Fab binding, we cannot attribute this to binding of MPV458 and MPV465 as other Fabs also caused trimer dissociation of this construct. The MPV458-hMPV B2 F complex was subjected to crystallization screening and crystals were obtained in 0.5 M ammonium sulfate, 0.1 M sodium citrate tribasic dihydrate pH 5.6, and 1.0 M Lithium sulfate monohydrate. Crystals were harvested and X-ray diffraction data was collected, and the structure of the complex was determined to 3.1 Å (**Fig 3; S3 Table**). The asymmetric unit contained one hMPV F protomer with one MPV458-Fab molecule. hMPV F was observed in the pre-fusion conformation, although no trimeric structure was observed when viewing symmetry related partners (**S6 Fig**). MPV458 targets a unique epitope compared to previously discovered Pneumovirus antigenic sites. The primary epitope consists of a single alpha helix of amino acids 66–87 of the F2 region (**Fig 3A**). Compared to the hMPV F protein, MPV458 binds nearly perpendicular to the long axis of the F protein. Upon overlay with the previously determined X-ray crystal structure of pre-fusion hMPV F, it is clear the major epitope lies completely within the interface between two protomers of trimeric hMPV F (**Fig 3B**). This unusual epitope suggests the hMPV F protein is partially monomeric on the surface of the virion envelope or on hMPV infected cells, and/or substantial breathing of the hMPV F protein takes place to allow the antibody to bind and neutralize the virus. As mentioned earlier, MPV458 has an unusually short HCDR3 loop of just 8 amino acids. The HCDR3 and light chain CDR (LCDR) 3 are centered on the 66–87 helix region. Numerous hydrogen bonding events were clear in the electron density (**Fig 3C; Fig 3D; S7 Fig**). The HCDR3 interacts via Asp107 with Arg79 of hMPV F, while HCDR2 Asn64 and Ser63 interact with hMPV F Glu80 and Arg205, respectively (**Fig 3C**). The HCDR1 utilizes Arg36 to interact with hMPV F Glu70. The light chain LCDR3 has more hydrogen bonding events than the HCDR3, utilizing the backbone amino group of Leu114 to interact with hMPV F Thr83, Arg115 to hMPV F Asp87, and Asp108 to hMPV F Lys82 (**Fig 3D**). LCDR1 Arg37 interacts with hMPV F Asn57, which has an extended N-linked glycan motif. In addition, the LCDR2 Asp56 interacts with hMPV F Thr56. The Framework 3 loop of the light chain interacts with the glycan motif consisting of NAG-NAG-BMA with branched MAN residues off the BMA glycan, in which Tyr83 interacts with the extended MAN glycan, while the long-face of Tyr83 sits parallel to the extended glycan, suggesting a favorable interaction with the glycan motif.

### Functional characterization of the 66–87 helical epitope

**Fig 4:**
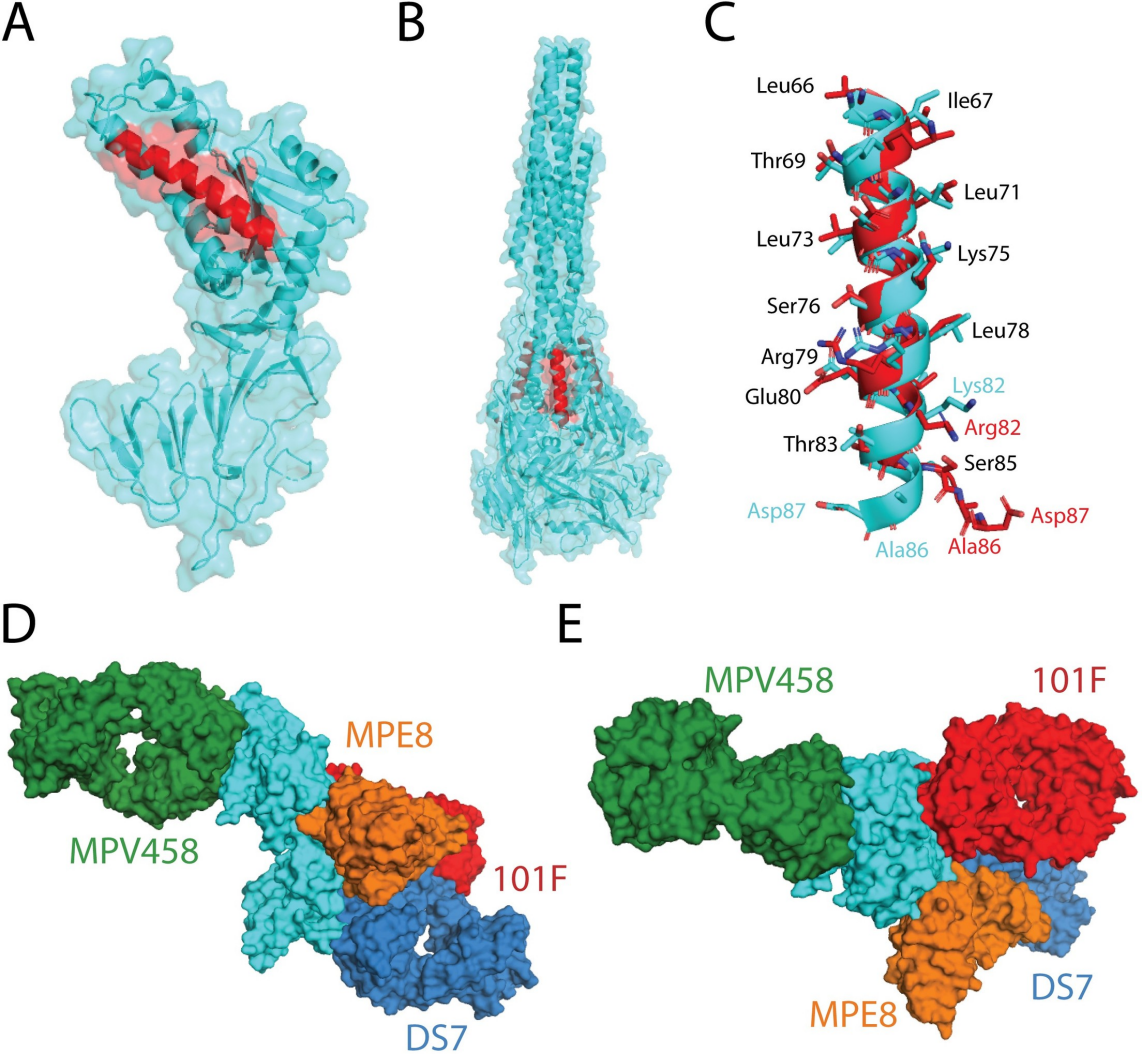
Structural comparison of the hMPV B2 F + MPV458 Fab complex. (A) The X-ray crystal structure of pre-fusion hMPV F is shown with the 66–87 epitope colored in red (PDB ID: 5WB0). (B) The corresponding 66–87 epitope is colored on the X-ray crystal structure of post-fusion hMPV F (5L1X). The 66–87 epitope is surface exposed on trimeric post-fusion hMPV F. (C) Structural overlay of the 66–87 region between pre-fusion (cyan) hMPV F from the hMPV B2 F + MPV458 Fab complex and post-fusion (red) hMPV F (PDB ID: 5L1X). Conserved amino acid residues between the B2 and A1 subgroups are listed in black, while residues that have mutations or shift positions are colored according to the corresponding structure. (D) Structural overlay of MPV458 on the hMPV F protein with previously structurally characterized hMPV F-specific mAbs. MPE8 (site III, orange) and 101F (site IV, red) were aligned onto hMPV F by aligning the corresponding RSV F residues onto hMPV F from the co-complex structures with RSV F (PDB ID: 5U68 and PBD ID: 3O45). DS7 was aligned from PDB 4DAG. (E) The structure overlay in (D) is rotated 90 degrees to view the hMPV F protein from the top down.

The 66–87 helix of hMPV F is structurally conserved in the pre-fusion and post-fusion conformations, although the helix is exposed on the outer surface in the trimeric post-fusion conformation (**Fig 4A; Fig 4B**). Upon overlay of the 66–87 region of the pre-fusion and post-fusion hMPV F proteins, residues 66–83 align well, while the helix breaks on post-fusion hMPV F at residues 84–87 (**Fig 4C)**. This sequence identity of the helix is highly conserved, as residues are identical between the A1 and B2 subgroups, except for a Lys82/Arg82 mutation. As MPV458 and MPV465 exhibited binding to post-fusion hMPV F constructs, we further examined binding by attempting to generate a complex between the Fab of MPV458 and trypsinized hMPV B2 F that was in the post-fusion conformation (**S2 Fig; S8 Fig**). No complex was observed as assessed by size exclusion chromatography, while the Fab of 101F readily formed a complex with the post-fusion hMPV F protein. This suggests that although binding is observed by ELISA, part of the epitope lies outside the 66–87 helix and is incomplete in the post-fusion conformation. Since the major epitope is focused on the single helix, we assessed binding by western blot to determine if MPV458 displayed binding to a linear conformation in the denatured hMPV F protein (**S9 Fig**). Binding to hMPV B2 F was analyzed using reduced and heated protein, and a nonreduced protein. MPV458 showed binding to all states of hMPV B2 F, while control mAbs 101F and MPE8 showed binding to only the nonreduced state. These data suggest the MPV458 epitope is at least partially linear. As the epitope for MPV458 lies within the trimer interface, the mechanism by which B cells recognize this epitope is unclear. To determine if the MPV458 epitope is exposed on the surface of hMPV infected cells, we performed flow cytometry using MPV458, MPE8, and a negative control pneumococcal-specific antibody (**S10 Fig**). Both MPV458 and MPE8 induced a fluorescent shift in hMPV infected cells, while the negative control mAb did not. This indicates the hMPV F protein is either in monomeric form on the surface of infected cells, or that hMPV F trimer exhibits breathing motion that allows for binding of MPV458. By comparing the binding sites with previously described hMPV F-specific mAbs that have been structurally characterized (MPE8, 101F, and DS7), the MPV458 epitope is distant from all three known antigenic sites (IV, DS7-site, and III), and lies on the opposite face of the monomeric hMPV F protein (**Fig 4D; Fig 4E**). This unique epitope was unexpected on the hMPV F protein, although intratrimeric epitopes have been observed on the influenza hemagglutinin protein [60–64]. However, these previously discovered influenza-specific mAbs have been nonneutralizing, and a subset were determined to function by disrupting the HA trimer and inhibiting cell-to-cell spread [60,64]. Evidence for pneumovirus F protein breathing was previously demonstrated for the RSV F protein, whereby the mAb CR9501 that binds at antigenic site V enhances opening of the pre-fusion RSV F protein [65], suggesting a similar process may occur for hMPV F.

## Discussion

Here we demonstrate a new class of neutralizing hMPV F-specific human mAbs. The mAbs are broadly reactive across all hMPV subgroups, and neutralize viruses from both hMPV genotypes. The mAbs were discovered to bind to a novel epitope by competition experiments with previously discovered rodent and human derived hMPV F-specific mAbs. The RSV F protein has at least two antigenic sites that are surface exposed on the head of the trimeric surface (antigenic sites Ø [66] and V [44,45]), however, such antigenic sites have not yet been identified for hMPV F, likely due to glycan shielding [38]. Furthermore, the X-ray crystal structure of one mAb, MPV458, was determined in complex with the hMPV F protein and solved to 3.1 Å. The structure revealed MPV458 binds at a newly defined epitope on the hMPV F protein defined by the alpha helical 66–87 amino acid region contained within the F2 fragment on pre-fusion hMPV F. This new epitope is the first defined on the head of the hMPV F protein as previous mAbs identified have targeted the lower half of the protein [43,50,52–55]. The new epitope is nearly completely contained within the pre-fusion trimeric interface of the hMPV F protein, which is a unique feature among previously discovered human mAbs to viral glycoproteins. Although the mAbs were shown to bind both predominantly pre-fusion and post-fusion conformations of the hMPV F protein, preferential binding to pre-fusion hMPV F was observed as evidenced by our attempts to complex MPV458 and MPV465 with post-fusion hMPV F. These data indicate that while the 66–87 epitope is present in both pre-fusion and post-fusion conformations, the complete structural epitope is present only on pre-fusion hMPV F, as several contacts outside of the 66–87 region were observed in our X-ray crystal structure. These additional epitope residues are rearranged in the post-fusion conformation. Human mAbs targeting the trimeric interface of the influenza HA protein have previously been discovered [60–64], yet these mAbs were nonneutralizing, unlike the mAbs described here, which are the first human mAbs discovered that bind within the trimeric interface of a viral glycoprotein and neutralize the virus. As epitopes at the trimeric interface have now been determined for influenza virus and hMPV, it is likely such epitopes are important mediators of viral immunity for other class I fusion viral glycoproteins.

The mechanism by which mAbs MPV458 and MPV465 neutralize hMPV remains to be determined. The mAbs could inhibit the transition of the hMPV F protein from the pre-fusion to the post-fusion conformation, which is likely the mechanism for the majority of antibodies targeting Pneumovirus fusion proteins. Alternatively, the mAbs could prevent infection by disrupting the trimeric structure of the hMPV F protein. Currently, we do not have reliable pre-fusion constructs that could be used to examine this hypothesis. It is clear that MPV458 binds to the surface of infected cells as demonstrated by our analysis by flow cytometry, although it is unclear if the mAb is binding to trimeric or monomeric hMPV F on the cell surface. Since the 66–87 epitope is hidden within the trimeric interface of the previously determined X-ray crystal structure of pre-fusion hMPV F [38], a mechanism must occur whereby the hMPV F protein motion facilitates exposure of the epitope for MPV458 binding, and indeed for initial naïve B cell recognition of this epitope since these mAbs were derived from seropositive human subjects. This motion, termed “breathing” has previously been demonstrated for the RSV F protein by identification of an alternative conformation of the RSV F protein, whereby the mAb CR9501 causes opening of pre-fusion RSV F trimers, and RSV F was also found to be both monomeric and trimeric on the surface of transfected HEK293F cells [65]. Furthermore, breathing of influenza and HIV glycoproteins has also been described [67,68], and mAbs to the HIV glycoprotein have been shown to destabilize the trimeric structure [69]. The mAb CR9501 targets antigenic site V of the RSV F protein, which was previously defined by the mAb hRSV90 [45]. mAbs to a similar antigenic site V epitope on the hMPV F protein have not been identified, and MPV458 targets an epitope on the opposite face of monomeric hMPV F.

Although we have identified a new antigenic site by isolating two mAbs from different donors, it remains unclear if such antibodies are a major part of the hMPV F humoral immune response. It also remains to be determined if mAbs such as MPV458 will protect against viral replication *in vivo*. Since the MPV458 epitope is partially linear, as evidenced by our binding studies to reduced hMPV F, a peptide-based vaccine based solely around this epitope may elicit neutralizing antibodies. Additionally, although MPV458 and MPV465 target a similar epitope based on epitope binning analysis, the binding properties to trimeric hMPV F are quite distinct. MPV458 shows binding to both monomeric and trimeric hMPV F constructs, while binding to trimeric hMPV F is completely eliminated for MPV465. Further structural analysis of the MPV465 epitope will delineate the differential binding properties. Our findings provide novel insights on the human antibody response to the hMPV F protein, and responses to viral glycoproteins. The X-ray crystal structure of the immune complex may guide the development of vaccines against hMPV. In addition, MPV458 can be potentially applied to the treatment and prevention of hMPV infection if prophylactic efficacy is demonstrated in animal challenge models.

## Methods

### Ethics statement

This study was approved by the University of Georgia Institutional Review Board as STUDY00005127. Healthy human donors were recruited to the University of Georgia Clinical and Translational Research Unit and written informed consent was obtained.

### Blood draws and PBMC isolation

After obtaining informed consent, 90 mL of blood was drawn by venipuncture into 9 heparin-coated tubes, and 10 mL of blood was collected into a serum separator tube. Peripheral blood mononuclear cells (PBMCs) were isolated from human donor blood samples using Ficoll-Histopaque density gradient centrifugation, and PBMCs were frozen in the liquid nitrogen vapor phase until further use.

### Production and purification of recombinant hMPV F proteins

Plasmids encoding cDNAs for hMPV F proteins listed in **S1 Table** were synthesized (GenScript) and cloned into the pcDNA3.1+ vector. The plasmids were expanded by transformation in *Escherichia coli* DH5α cells with 100 μg/mL of ampicillin (Thermo Fisher Scientific) for selection. Plasmids were purified using the EZNA plasmid maxi kit (Omega BioTek), according to the manufacturer’s protocol. To generate stable cell lines that express hMPV B2 F, hMPV B2 F-GCN4, and hMPV F 130-BV, Expi293F (Thermo Fisher Scientific) cells were plated into a 12 well plate (4 x 10^5^ per well) with 1 mL of growth medium (Dulbecco's Modified Eagle Medium (Corning), 10% fetal bovine serum (Corning)) 1 day before transfection. For each milliliter of transfection, 1 μg of plasmid DNA was mixed with 4 μg of 25,000-molecular-weight polyethylenimine (PEI; PolySciences Inc.) in 66.67 μl Opti-MEM cell culture medium (Gibco). After 30 min, the DNA-PEI mixture was added to HEK293F cells in Opti-MEM. After 3 to 4 days, 20 μl of cell culture supernatant was used for western blot to determine protein expression. Then, the culture medium was replaced with 1 mL growth medium supplemented with G418 (Geneticin; VWR) antibiotic to a final concentration of 250 μg/mL. After 2–3 days, HEK293F cells were resuspended with the growth medium supplemented with G418, and expanded to a 25-cm^2^ cell culture flask. Cells were trypsinized once they reach 80–90% confluency and further expanded to a 75-cm^2^ cell culture flask. Again, at 80–90% confluency, trypsinized the cells were transferred to a 250 mL flask in 100 mL Freestyle293 medium (Gibco) supplemented with G418 and cultured in shaking incubator at 37°C with 5% CO_2_. For protein expression and purification, the stable cell lines were expanded in 500 mL of Freestyle293 medium supplemented with G418. The remaining constructs are expressed by transient transfection of Expi293F cells. After 5 to 7 days, the cultures were centrifuged to pellet the cells, and the supernatants were filtered through a 0.45-μm sterile filter. Recombinant proteins were purified directly from the filtered culture supernatants using HisTrap Excel columns (GE Healthcare Life Sciences). Each column was stored in 20% ethanol and washed with 5 column volumes (CV) of wash buffer (20 mM Tris pH 7.5, 500 mM NaCl, and 20 mM imidazole) before loading samples onto the column. After sample application, columns were washed with 10 CV of wash buffer. Proteins were eluted from the column with 6 CV of elution buffer (20 mM Tris pH 7.5, 500 mM NaCl, and 250 mM imidazole). Proteins were concentrated and buffer exchanged into phosphate buffered saline (PBS) using Amicon Ultra-15 centrifugal filter units with a 30-kDa cutoff (Millipore Sigma).

### Trypsinization of hMPV F

In order to generate homogeneous cleaved trimeric hMPV F, TPCK (L-1-tosylamido-2-phenylethyl chloromethyl ketone)-trypsin (Thermo Scientific) was dissolved in double-distilled water (ddH_2_O) at 2 mg/mL. Purified hMPV B2 F was incubated with 5 TAME (p-toluene-sulfonyl-L-arginine methyl ester) units/mg of TPCK-trypsin for 1 hr at 37°C. Trimeric and monomeric hMPV B2 F proteins were purified from the digestion reaction mixture by size exclusion chromatography on a Superdex S200, 16/600 column (GE Healthcare Life Sciences) in column buffer (50 mM Tris pH 7.5, and 100 mM NaCl). Trimeric hMPV B2 F protein was identified by a shift in the elution profile from monomeric hMPV B2 F protein. The fractions containing the trimers and monomers were concentrated using 30-kDa Spin-X UF concentrators (Corning). To obtain fully post-fusion hMPV F, samples were heated at 55°C for 20 minutes to induce conversion of remaining pre-fusion hMPV F proteins to the post-fusion conformation.

### Negative-stain electron microscopy analysis

All samples were purified by size exclusion chromatography on a Superdex S200, 16/600 column (GE Healthcare Life Sciences) in column buffer before they were applied on grids. Carbon-coated copper grids (Electron Microscopy Sciences) were overlaid with 5 μl of protein solutions (10 μg/mL) for 3 min. The grid was washed in water twice and then stained with 0.75% uranyl formate for 1 min. Negative-stain electron micrographs were acquired using a JEOL JEM1011 transmission electron microscope equipped with a high-contrast 2K-by-2K AMT midmount digital camera.

### Generation of hMPV F-specific hybridomas

For hybridoma generation, 10 million peripheral blood mononuclear cells purified from the blood of human donors were mixed with 8 million previously frozen and gamma irradiated NIH 3T3 cells modified to express human CD40L, human interleukin-21 (IL-21), and human BAFF [54] in 80 mL StemCell medium A (StemCell Technologies) containing 6.3 μg/mL of CpG (phosphorothioate-modified oligodeoxynucleotide ZOEZOEZZZZZOEEZOEZZZT; Invitrogen) and 1 μg/mL of cyclosporine A (Millipore-Sigma). The mixture of cells was plated in four 96-well plates at 200 μl per well in StemCell medium A. After 6 days, culture supernatants were screened by ELISA for binding to recombinant hMPV B2 F protein, and cells from positive wells were electrofused as previously described [54]. Cells from each cuvette were resuspended in 20 mL StemCell medium A containing 1× HAT (hypoxanthine-aminopterin-thymidine; Sigma-Aldrich), 0.2× HT (hypoxanthine-thymidine; Corning), and 0.3 μg/mL ouabain (Thermo Fisher Scientific) and plated at 50 μl per well in a 384-well plate. After 7 days, cells were fed with 25 μl of StemCell medium A. The supernatant of hybridomas were screened after 2 weeks for antibody production by ELISA, and cells from wells with reactive supernatants were expanded to 48-well plates for 1 week in 0.5 mL of StemCell medium E (StemCell Technologies), before being screened again by ELISA. Positive hybridomas were then subjected to single-cell fluorescence-activated sorting into 384-well plates containing 75% of StemCell medium A plus 25% of StemCell medium E. Two weeks after cell sorting, hybridomas were screened by ELISA before further expansion of wells containing hMPV F-specific hybridomas.

### Human mAb and Fab production and purification

For recombinant mAbs, plasmids encoding cDNAs for the heavy and light chain sequences of 101F [70], MPE8 [51], and DS7 [49] were synthesized (GenScript), and cloned into vectors encoding human IgG1 and lambda or kappa light chain constant regions, respectively. mAbs were obtained by transfection of plasmids into Expi293F cells as described above. For hybridoma-derived mAbs, hybridoma cell lines were expanded in StemCell medium A until 80% confluent in 75-cm^2^ flasks. Cells from one 75-cm^2^ cell culture flask were collected with a cell scraper and expanded to four 225-cm^2^ cell culture flasks in serum-free medium (Hybridoma-SFM; Thermo Fisher Scientific). Recombinant cultures from transfection were stopped after 5 to 7 days, and hybridoma cultures were stopped after 30 days. Culture supernatants from both approaches were filtered using 0.45 μm filters to remove cell debris. mAbs were purified directly from culture supernatants using HiTrap protein G columns (GE Healthcare Life Sciences) according to the manufacturer’s protocol. To obtain Fab fragments, papain digestion was performed using the Pierce Fab preparation kit (Thermo Fisher Scientific) according to the manufacturer’s protocol. Fab fragments were purified by removing IgG and Fc contaminants using a HiTrap MabSelectSure (GE Healthcare Life Sciences) column according to the manufacturer’s protocol.

### Isotype determination for human mAbs

For determination of mAb isotypes, 96-well Immulon HB 4× ELISA plates (Thermo Fisher Scientific) were coated with 2 μg/mL of each mAb in PBS (duplicate wells for each sample). The plates were incubated at 4°C overnight and then washed once with water. Plates were blocked with blocking buffer (2% nonfat milk, 2% goat serum in PBS with 0.05% Tween 20 (PBS-T)) and then incubated for 1 hr at room temperature. After incubation, the plates were washed three times with water. Isotype-specific antibodies obtained from Southern Biotech (goat anti-human kappa-alkaline phosphatase [AP] [catalog number 100244–340], goat anti-human lambda-AP [catalog number 100244–376], mouse anti-human IgG1 [Fc]-AP [catalog number 100245714], mouse anti-human IgG2 [Fc]-AP [catalog number 100245–734], mouse anti-human IgG3 [hinge]-AP [catalog number 100245–824], and mouse anti-human IgG4 [Fc]-AP [catalog number 100245–812]) were diluted 1:1,000 in blocking buffer, and 50 μl of each solution was added to the respective wells. Plates were incubated for 1 hr at room temperature and then washed five times with PBS-T. The PNPP substrate was prepared at 1 mg/mL in substrate buffer (1 M Tris base, 0.5 mM MgCl_2_, pH 9.8), and 100 μl of this solution was added to each well. Plates were incubated for 1 hr at room temperature and read at 405 nm on a BioTek plate reader.

### RT-PCR for hybridoma mAb variable gamma chain and variable light chain

RNA was isolated from expanded hybridoma cells using the ENZA total RNA kit (Omega BioTek) according to the manufacturer’s protocol. A Qiagen OneStep RT-PCR kit was used for cDNA synthesis and PCR amplification. For RT-PCR, 50 μl reaction mixtures were designed with the following final concentrations: 1× Qiagen OneStep RT-PCR buffer, 400 μM deoxynucleoside triphosphate (dNTP) mix, 0.6 μM primer mix, 2 μl of Qiagen OneStep RT-PCR enzyme mix, 1 μg total of the template RNA, and RNase-free water. Three separate sets of primer mixes were used: gamma, kappa and lambda forward and reverse primers as previously described [71]. The RT-PCR was performed in a thermocycler with the following program: 30 min at 50°C, 15 min at 95°C, and then a 3-step cycle with 30 repeats of denaturation for 30 s at 94°C, annealing for 30 s at 50°C, and extension for 1 min at 72°C, followed by 10 min of final extension at 72°C. Samples were analyzed by agarose gel electrophoresis and purified PCR products (ENZA cycle pure kit; Omega Biotek) were cloned into the pCR2.1 vector using the Original TA cloning kit (Thermo Fisher Scientific) according to the manufacturer’s protocol. Plasmids were purified from positive DH5α colonies with ENZA plasmid DNA mini kit (Omega Biotek) and submitted to Genewiz for sequencing. Sequences were analyzed using IMGT/V-Quest [72]. For MPV458, 2 × 10^6^ of hybridoma cells were sent to GenScript for antibody variable domain sequencing.

### Enzyme-linked immunosorbent assay for binding to hMPV F proteins

For recombinant protein capture ELISAs, 384-well plates (Greiner Bio-One) were treated with 2 μg/ml of antigen in PBS for 1 hr at 37°C or overnight at 4°C. Following this, plates were washed once with water before blocking for 1 hr with 2% blocking buffer. Primary mAbs or culture supernatants were applied to wells for 1 hr following three washes with water. Plates were washed with water three times before applying 25 μl of secondary antibody (goat anti-human IgG Fc; Meridian Life Science) at a dilution of 1:4,000 in blocking solution. After incubation for 1 hr, the plates were washed five times with PBS-T, and 25 μl of a PNPP (p-nitrophenyl phosphate) solution (1 mg/ml PNPP in 1 M Tris base) was added to each well. The plates were incubated at room temperature for 1 hr before reading the optical density at 405 nm on a BioTek plate reader. Binding assay data were analyzed in GraphPad Prism using a nonlinear regression curve fit and the log(agonist)-versus-response function to calculate the binding EC_50_ values.

### Experimental setup for biolayer interferometry

For all biosensors, an initial baseline in running buffer (PBS, 0.5% bovine serum albumin [BSA], 0.05% Tween 20, 0.04% thimerosal) was obtained. For epitope mapping, 100 μg/mL of His-tagged hMPV 130-BV F [38] protein was immobilized on anti-penta-HIS biosensor tips (FortéBio) for 120 s. The baseline signal was measured again for 60 s before biosensor tips were immersed into wells containing 100 μg/mL of primary antibody for 300 s. Following this, biosensors were immersed into wells containing 100 μg/mL of a second mAb for 300 s. Percent binding of the second mAb in the presence of the first mAb was determined by comparing the maximal signal of the second mAb after the first mAb was added to the maximum signal of the second mAb alone. mAbs were considered noncompeting if maximum binding of the second mAb was ≥66% of its uncompeted binding. A level of between 33% and 66% of its uncompeted binding was considered intermediate competition, and ≤33% was considered competition. For affinity studies, monomeric hMPV B2 F protein was purified by size exclusion chromatography, biotinylated, and loaded onto streptavidin biosensors at 10 μg/mL for 10 s, and decreasing concentrations of Fabs were analyzed for binding by association for 300 s and dissociation for 600 s. Octet data analysis software was used to analyze the data. Values for reference wells containing no antibody were subtracted from the data and were fit to a 1:1 binding model. Binding curves were independently graphed in GraphPad Prism for data visualization.

### hMPV plaque neutralization assay

LLC-MK2 cells (ATCC CCL-7) were maintained in Opti-MEM (Thermo Fisher Scientific) supplemented with 2% heat inactivated fetal bovine serum and grown in 225-cm^2^ flask at 37°C in a 5% CO_2_ incubator. Two days prior to neutralization assays, cells were trypsinized and diluted in Opti-MEM at 80,000 cells/mL, 0.5 mL of cells were seeded into 24-well plates. On the day of the experiment, serially diluted mAbs isolated from hybridoma supernatants were incubated 1:1 with a suspension of infectious hMPV B2 strain TN/93-32 or hMPV A2 strain CAN/97-83 for 1 hr. Following this, cells were washed twice with PBS to remove serum, and inoculated with 50 μl of the antibody-virus mixture for 1 hr with rocking at room temperature. Cells were then overlaid with 1 mL of 0.75% methylcellulose dissolved in Opti-MEM supplemented with 5 μg/mL trypsin-EDTA and 100 μg/mL CaCl_2_. Cells were incubated for 4 days, after which the cells were fixed with 10% neutral buffered formalin. The cell monolayers were then blocked with blocking buffer (2% nonfat milk supplemented with 2% goat serum in PBS-T) for 1 hr. The plates were washed with water, and 200 μl of mouse anti-hMPV N primary antibody (catalog number C01851M; Meridian Biosciences) diluted 1:1,000 in blocking buffer was added to each well, and the plates were incubated for 1 hr. The plates were then washed three times with water, after which 200 μl of goat anti-mouse IgG-horseradish peroxidase (HRP) secondary antibody (catalog number 5220–0286; SeraCare) diluted 1:1,000 in blocking solution was added to each well for 1 hr. Plates were then washed five times with water, and 200 μl of TrueBlue peroxidase substrate (SeraCare) was added to each well. Plates were incubated until plaques were clearly visible. Plaques were counted by hand under a stereomicroscope and compared to a virus-only control, and the data were analyzed in GraphPad Prism using a nonlinear regression curve fit and the log(inhibitor)-versus-response function to calculate the IC_50_ values.

### Western blot

Protein samples in reducing condition were mixed with loading buffer containing β-mercaptoethanol and heated at 96°C for 10 min before loading on 4–12% Bis-Tris Plus gels (Invitrogen). Samples in non-reducing conditions were diluted in loading buffer without any other treatment. Samples were transferred to PVDF membranes via iBlot system (Invitrogen) and blocked with 5% blocking buffer (5% nonfat milk, 2% goat serum in PBS-T) at 4°C overnight. Primary antibodies were diluted at 0.5 μg/mL in PBS-T and HRP-conjugated goat anti-human secondary antibody was diluted at 1:10,000 in PBS-T. Both incubations were 1 hr at room temperature with a 5x PBS-T wash in between. Substrate (Pierce ECL Western Blotting Substrate, Thermo Scientific) was added immediately before the image was taken with ChemiDoc Imaging System (BioRad).

### Crystallization and structure determination of the MPV458 Fab + B2 F complex

To generate the complex of hMPV B2 F + MPV458 Fab complex, purified trypsinized B2 F trimer was added to MPV458 Fab at a 1:2 molar ratio and incubate at 4°C overnight. To crystallize the complex, the sample was subjected to size exclusion chromatography (S200, 16/300, GE Healthcare Life Sciences) in 50 mM Tris pH 7.5, 100 mM NaCl. The fractions containing the complex were concentrated to 15 mg/mL and crystallization trials were prepared on a TTP LabTech Mosquito Robot in sitting-drop MRC-2 plates (Hampton Research) using several commercially available crystallization screens. Crystals were obtained in the Crystal Screen HT (Hampton Research) in condition F3 (0.5 M Ammonium sulfate, 0.1 M Sodium citrate tribasic dihydrate pH 5.6, 1.0 M Lithium sulfate monohydrate). Crystals were harvested and cryo-protected with 30% glycerol in the mother liquor before being flash frozen in liquid nitrogen. X-ray diffraction data were collected at the Advanced Photon Source SER-CAT beamline 21-ID-D. Data were indexed and scaled using XDS [73]. A molecular replacement solution was obtained in Phaser [74] using the hMPV pre-fusion F structure (PDB 5WB0) and the Fab structure (PDB 4Q9Q). The structure of the complex was completed by manually building in COOT [75] followed by subsequent rounds of manual rebuilding and refinement in Phenix [74]. The data collection and refinement statistics are shown in S3 Table.

### Flow cytometry of hMPV infected LLC-MK2 cells

LLC-MK2 cells were cultured in 75-cm^2^ flask at 80–90% confluency, and then infected with hMPV (CAN/97-83) at 0.1 MOI in Opti-MEM containing 100 μg/mL CaCl_2_ and 5 μg/mL trypsin-EDTA. After 48 hrs, cells were washed twice with PBS and digested with Versene (Gibco) at 37°C for 40–50 minutes. Cells were washed once with PBS then transferred to 1.5 mL tubes, pelleted and resuspended in 1 mL FACS buffer (PBS containing 5% FBS, inactivated 2% Human serum, inactivated 2% goat serum, 2 mM EDTA pH 8.0, 10% sodium azide) and incubated for 30 min to block Fc receptors. Cells were washed three times with PBS, then aliquoted in a 96 well U bottom plate for antibody staining. Mouse anti-human IgG Fc APC (BioLegend, 409306) was used for secondary antibody staining. Stained cells were fixed in 4% paraformaldehyde and data was collected with Beckman Coulter CytoFLEX flow cytometer. Data were analyzed in FlowJo.

## Supporting information

S1 Fig Representative affinity traces for competitive biolayer interferometry. In (A), hMPV F protein loaded biosensors are exposed to each mAb displayed in the legend. In (B) and (C), the biosensors loaded with the first mAb are exposed to MPV458 (B) or MPV465 (C). A decrease in signal was observed when attempting to load 458 or 465 in the presence of biosensors already loaded with MPV458 or MPV465. No competition between MPV458 or MPV465 and other control mAbs was observed, and competition was observed between MPV458 and MPV465.
(TIF)

S2 Fig Negative-stain electron micrograph of purified hMPV B2 F prior to treatment with trypsin. A mixture of pre-fusion trimers, post-fusion trimers, and monomeric protein was observed.
(TIF)

S3 Fig Negative-stain electron micrograph of hMPV B2 F after treatment with trypsin and heating. Trimeric protein was purified by size exclusion chromatography before being subjected to negative-stain electron microscopy.
(TIF)

S4 Fig Chromatograms of size exclusion chromatography of hMPV B2 F and hMPV B2 F + Fab complexes. Trypsinization of hMPV B2 F generates homogeneous trimeric and monomeric peaks. Complexing trimeric hMPV B2 F with Fabs of MPV458 or MPV465 generates monomeric F-Fab complexes and excess Fabs. Data are representative of at least two independent experiments.
(TIF)

S5 Fig SDS-PAGE of hMPV F-Fab complexes. Excess Fab was added to trypsinized hMPV B2 F, and the complexes were purified by size exclusion chromatography. The peak corresponding to the hMPV B2 F-458Fab complex in Figure S4 is shown in lane 4, while the excess Fab is in lane 1. All samples were run on the same SDS-PAGE gel and each cropped image is aligned with the ladder as in the original gel image.
(TIF)

S6 Fig Symmetry-related partners in the hMPV B2 F + MPV458 Fab complex. **No trimeric structure was observed for the hMPV F protein.** The hMPV F protein is shown in cyan, while the MPV458 Fab is shown in green.
(TIF)

S7 Fig Representative image of the electron density map surrounding the hMPV B2 F + MPV458 X-ray crystal structure. The image was made in COOT and the map level rmsd is set to 2.45.
(TIF)

S8 Fig Chromatographs from size exclusion chromatography of post-fusion hMPV B2 F in complex with 101F and MPV458. No complexes were observed with MPV458, while 101F readily formed complexes with hMPV F.
(TIF)

S9 Fig Western blot analysis of MPV458 binding to the 66–87 epitope. A panel of hMPV F protein constructs were subjected to SDS-PAGE separation before transfer to a PVDF membrane. Specific mAbs listed above each panel were used as primary antibodies. MPV458 bound to all constructs tested including boiled samples, while MPE8 and 101F bound only to samples with limited treatment.
(TIF)

S10 Fig Flow cytometric analysis of hMPV F infected LLC-MK2 cells. Twenty-four hours after infection, cells were harvested and stained with mAbs indicated. MPV458 and MPE8 induced a fluorescent shift in infected cells as compared to the pneumococcal-specific mAb PspA16.
(TIF)

S1 Table Properties and sequences of recombinant hMPV F protein constructs used in this study. (DOCX)

S2 Table IMGT V-QUEST Analysis of MPV458 and MPV465. (DOCX)

S3 Table Data collection and refinement statistics. (DOCX)
