# Supplementary figures and images for: Antibody recognition of the Pneumovirus fusion protein trimer interface
Source: PLoS Pathog. 2020 Oct 9;16(10):e1008942. doi: 10.1371/journal.ppat.1008942 (PMC7598476; doi:10.1371/journal.ppat.1008942)

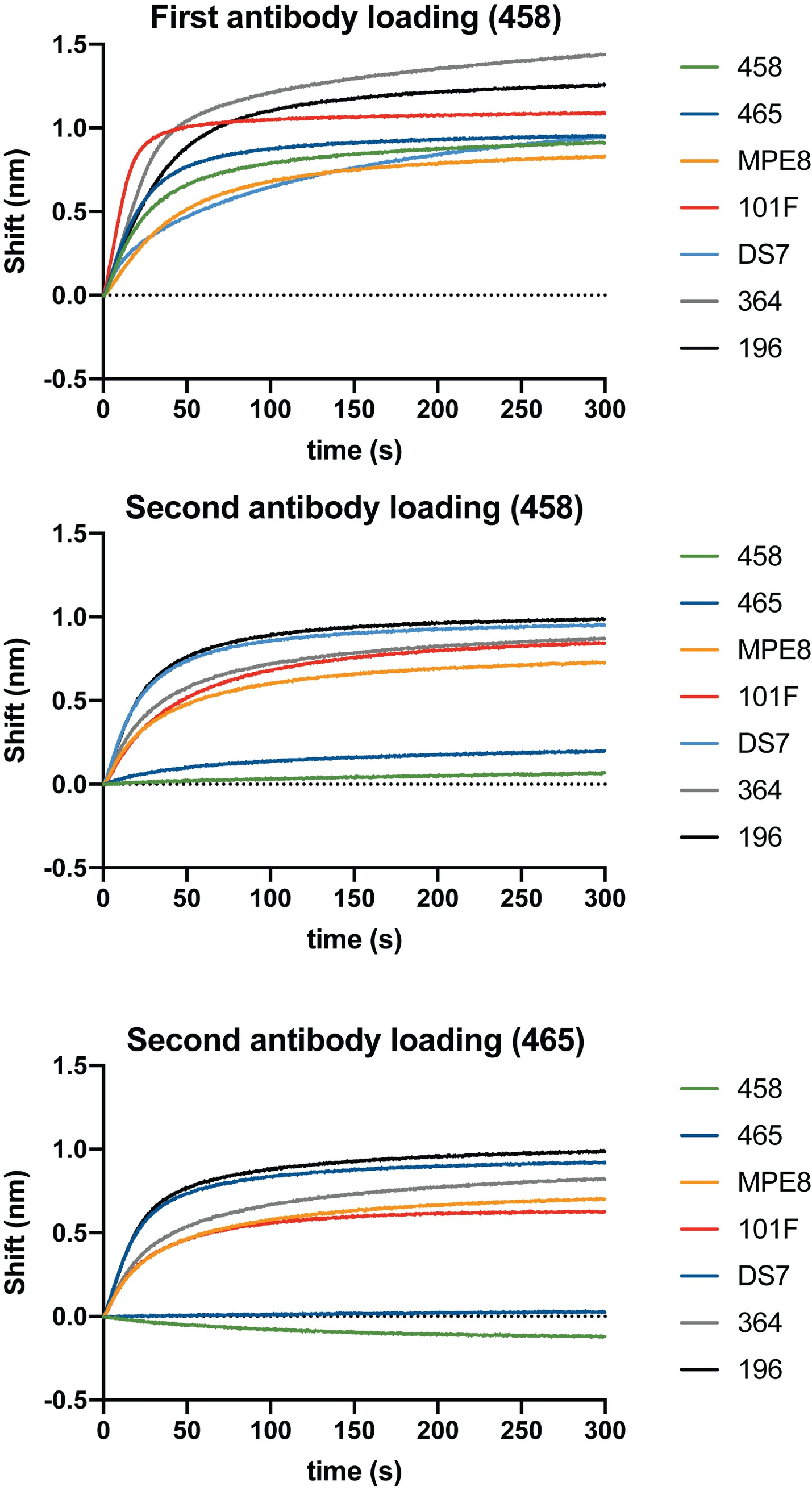

Supplement: S1 Fig — In (A), hMPV F protein loaded biosensors are exposed to each mAb displayed in the legend. In (B) and (C), the biosensors loaded with the first mAb are exposed to MPV458 (B) or MPV465 (C). A decrease in signal was observed when attempting to load 458 or 465 in the presence of biosensors already loaded with MPV458 or MPV465. No competition between MPV458 or MPV465 and other control mAbs was observed, and competition was observed between MPV458 and MPV465. (TIF) [file ppat.1008942.s001.tif]

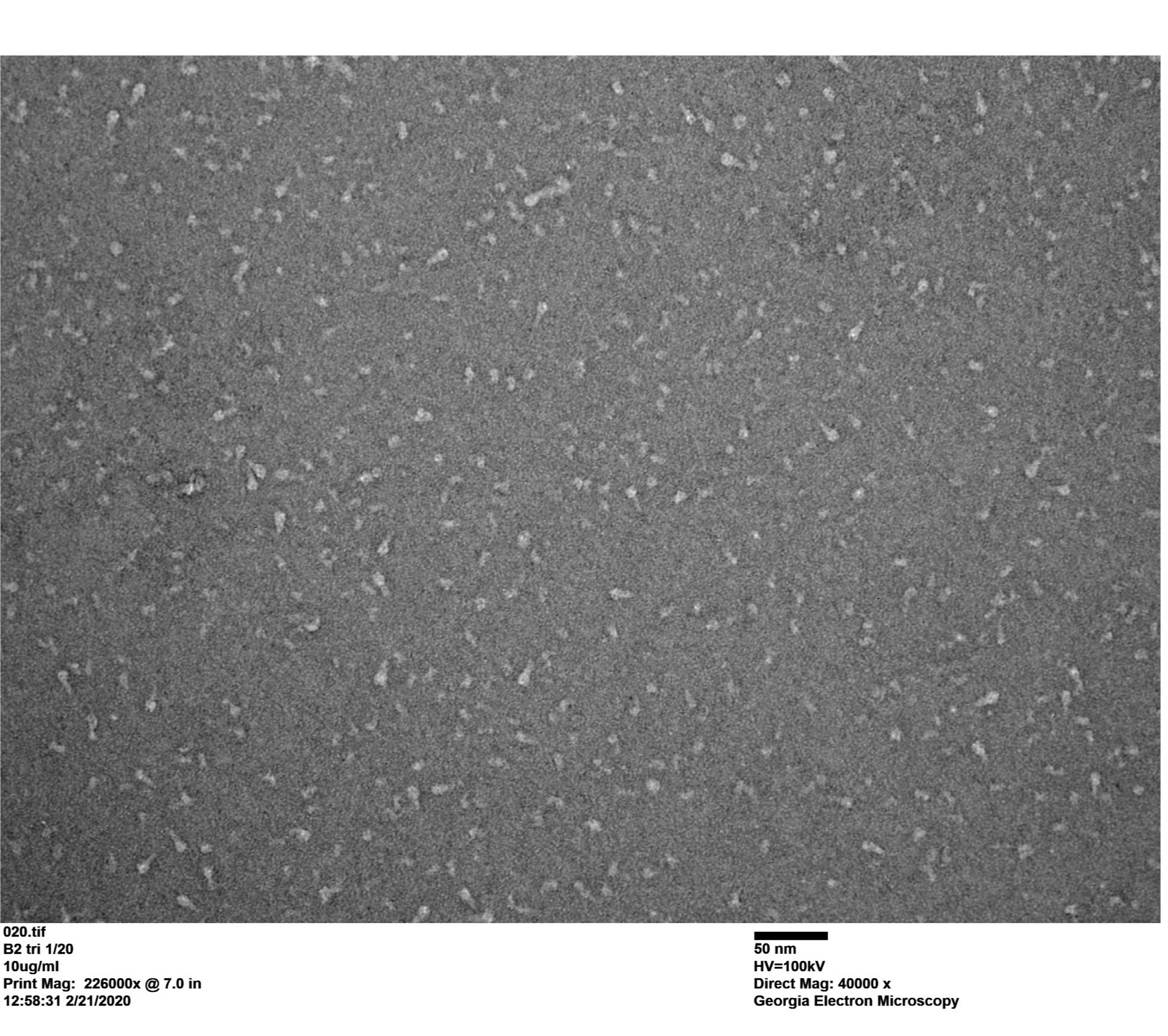

Supplement: S2 Fig — A mixture of pre-fusion trimers, post-fusion trimers, and monomeric protein was observed. (TIF) [file ppat.1008942.s002.tif]

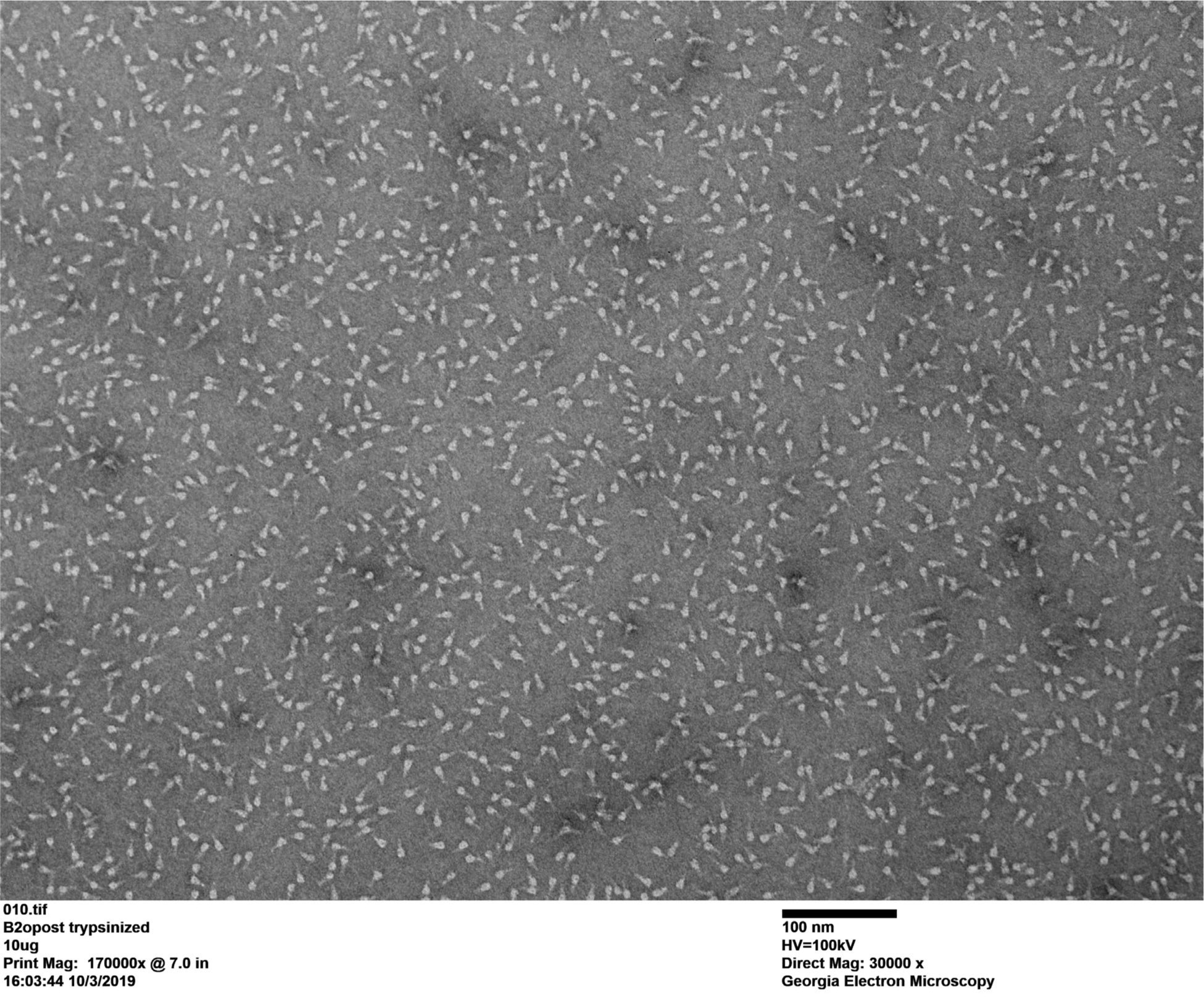

Supplement: S3 Fig — Trimeric protein was purified by size exclusion chromatography before being subjected to negative-stain electron microscopy. (TIF) [file ppat.1008942.s003.tif]

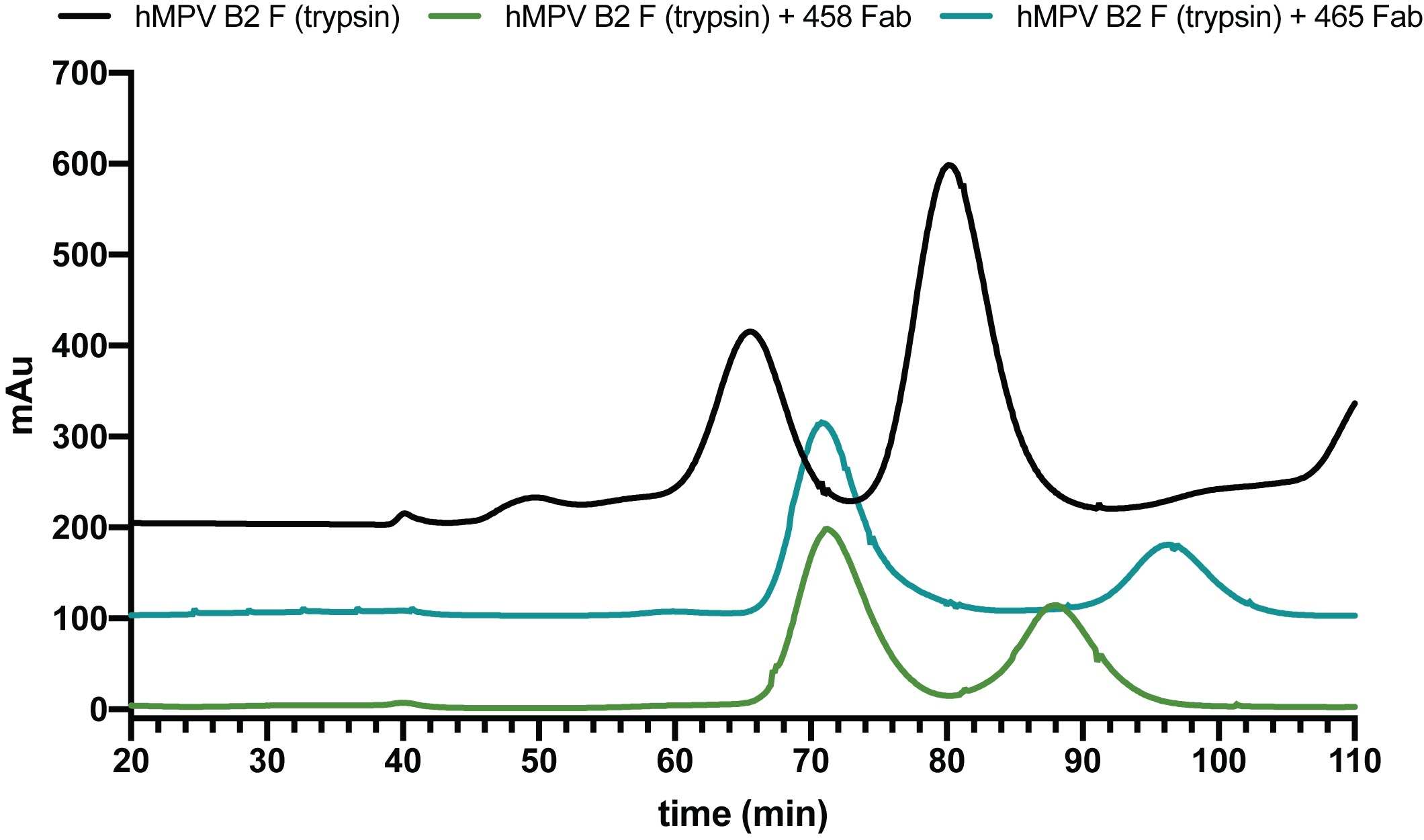

Supplement: S4 Fig — Trypsinization of hMPV B2 F generates homogeneous trimeric and monomeric peaks. Complexing trimeric hMPV B2 F with Fabs of MPV458 or MPV465 generates monomeric F-Fab complexes and excess Fabs. Data are representative of at least two independent experiments. (TIF) [file ppat.1008942.s004.tif]

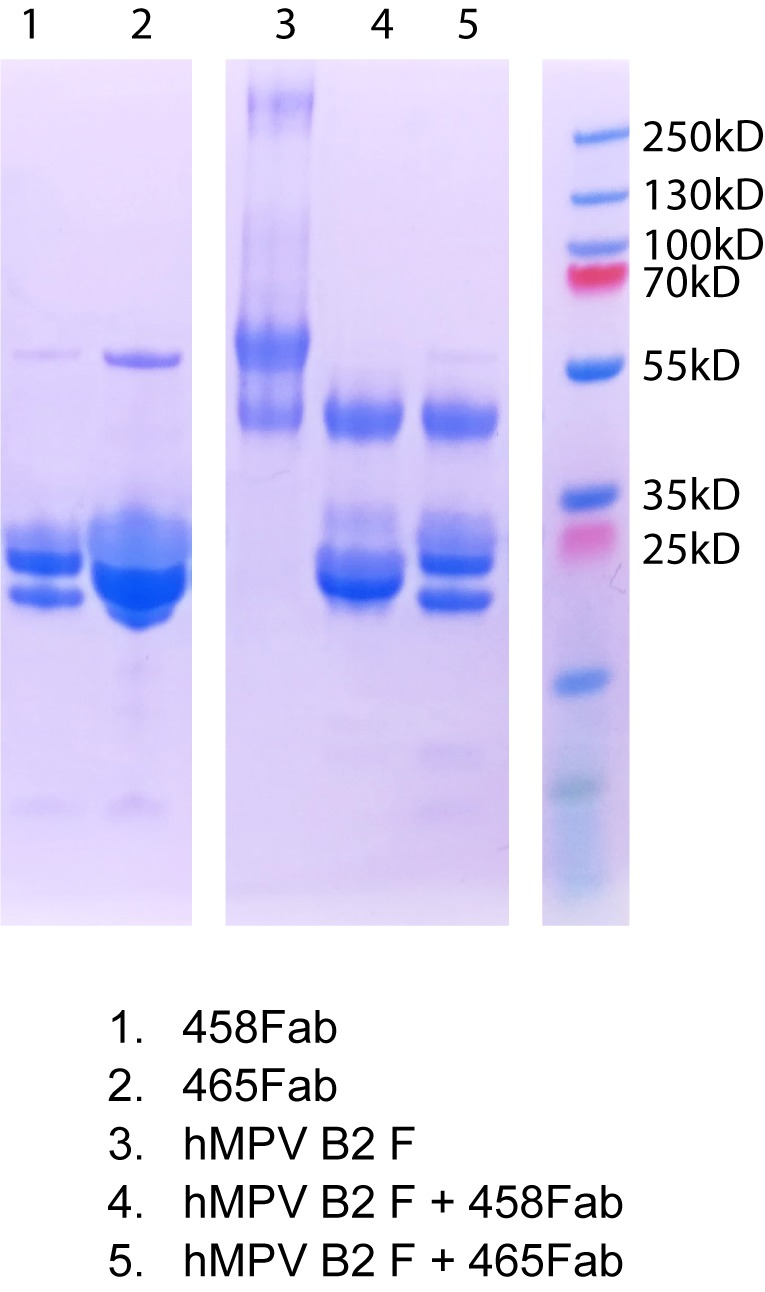

Supplement: S5 Fig — Excess Fab was added to trypsinized hMPV B2 F, and the complexes were purified by size exclusion chromatography. The peak corresponding to the hMPV B2 F-458Fab complex in Figure S4 is shown in lane 4, while the excess Fab is in lane 1. All samples were run on the same SDS-PAGE gel and each cropped image is aligned with the ladder as in the original gel image. (TIF) [file ppat.1008942.s005.tif]

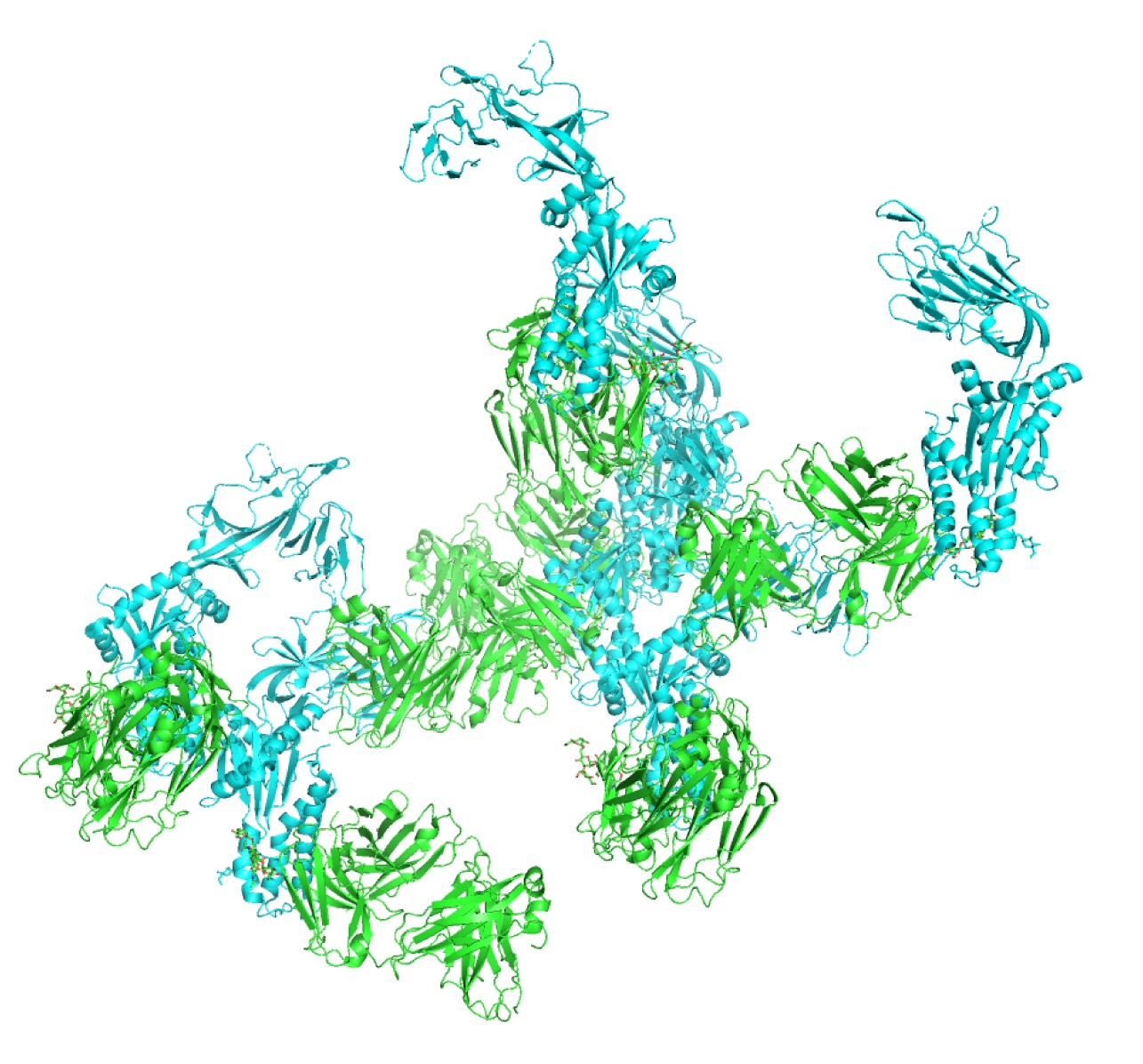

Supplement: S6 Fig — No trimeric structure was observed for the hMPV F protein. The hMPV F protein is shown in cyan, while the MPV458 Fab is shown in green. (TIF) [file ppat.1008942.s006.tif]

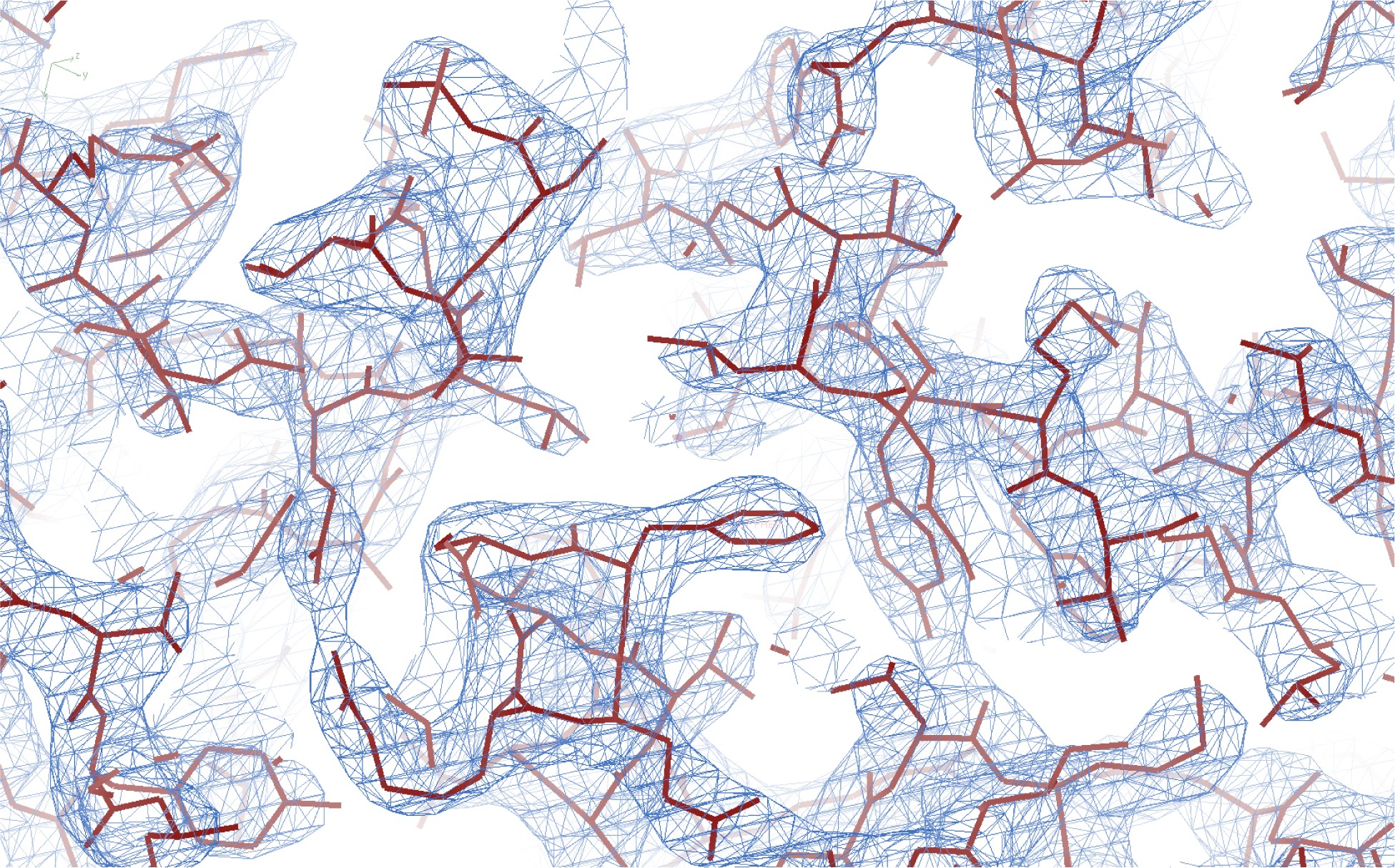

Supplement: S7 Fig — The image was made in COOT and the map level rmsd is set to 2.45. (TIF) [file ppat.1008942.s007.tif]

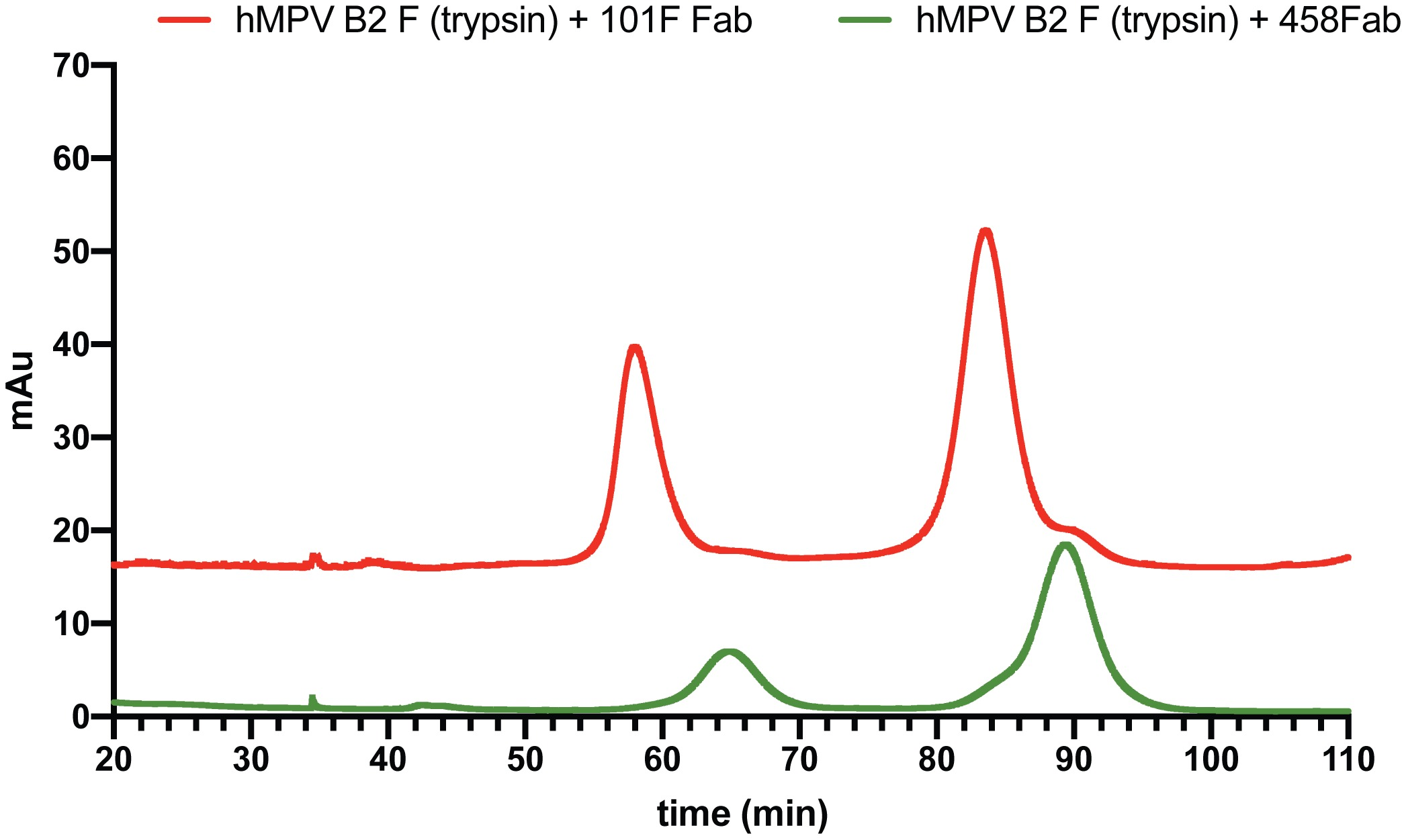

Supplement: S8 Fig — No complexes were observed with MPV458, while 101F readily formed complexes with hMPV F. (TIF) [file ppat.1008942.s008.tif]

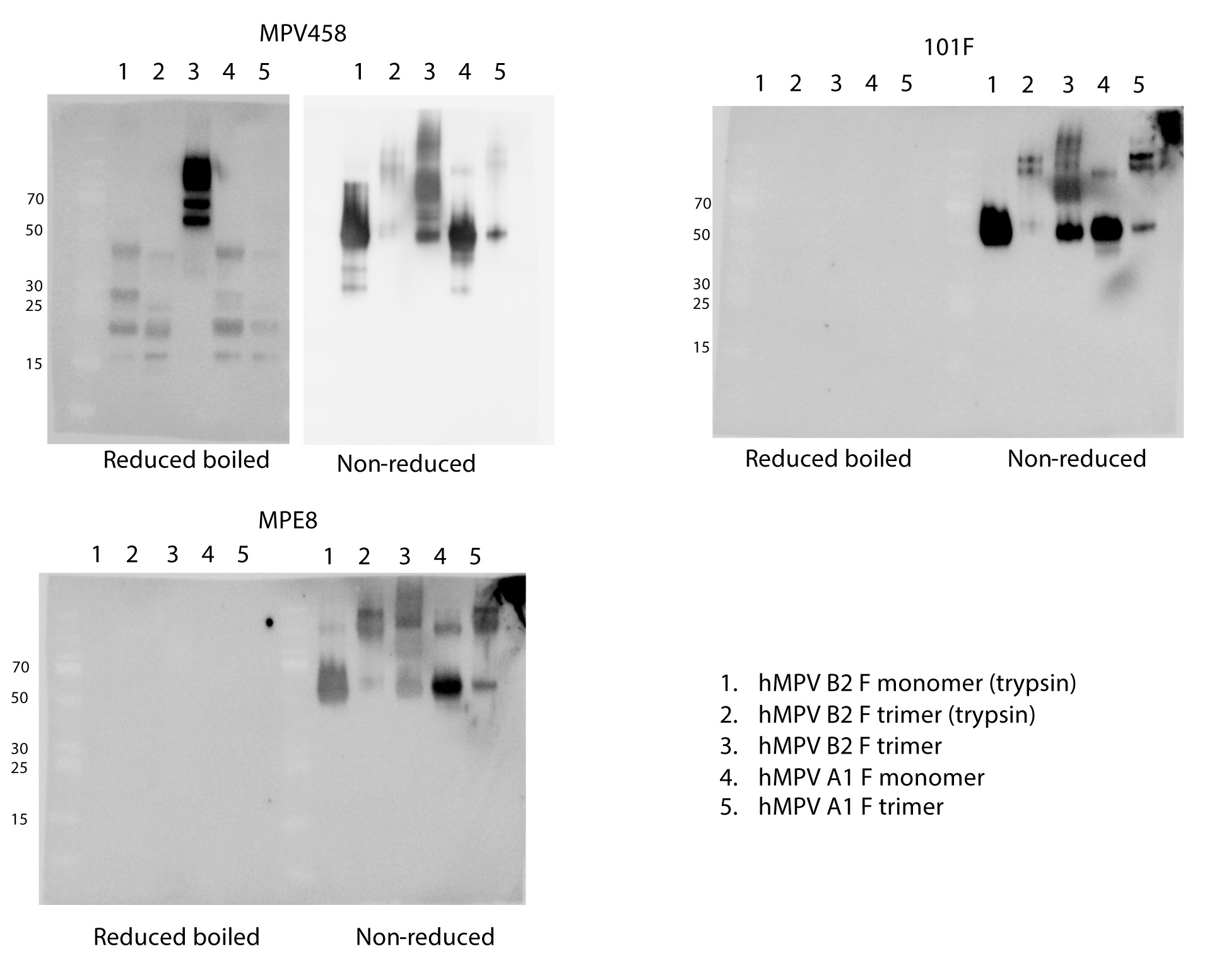

Supplement: S9 Fig — A panel of hMPV F protein constructs were subjected to SDS-PAGE separation before transfer to a PVDF membrane. Specific mAbs listed above each panel were used as primary antibodies. MPV458 bound to all constructs tested including boiled samples, while MPE8 and 101F bound only to samples with limited treatment. (TIF) [file ppat.1008942.s009.tif]

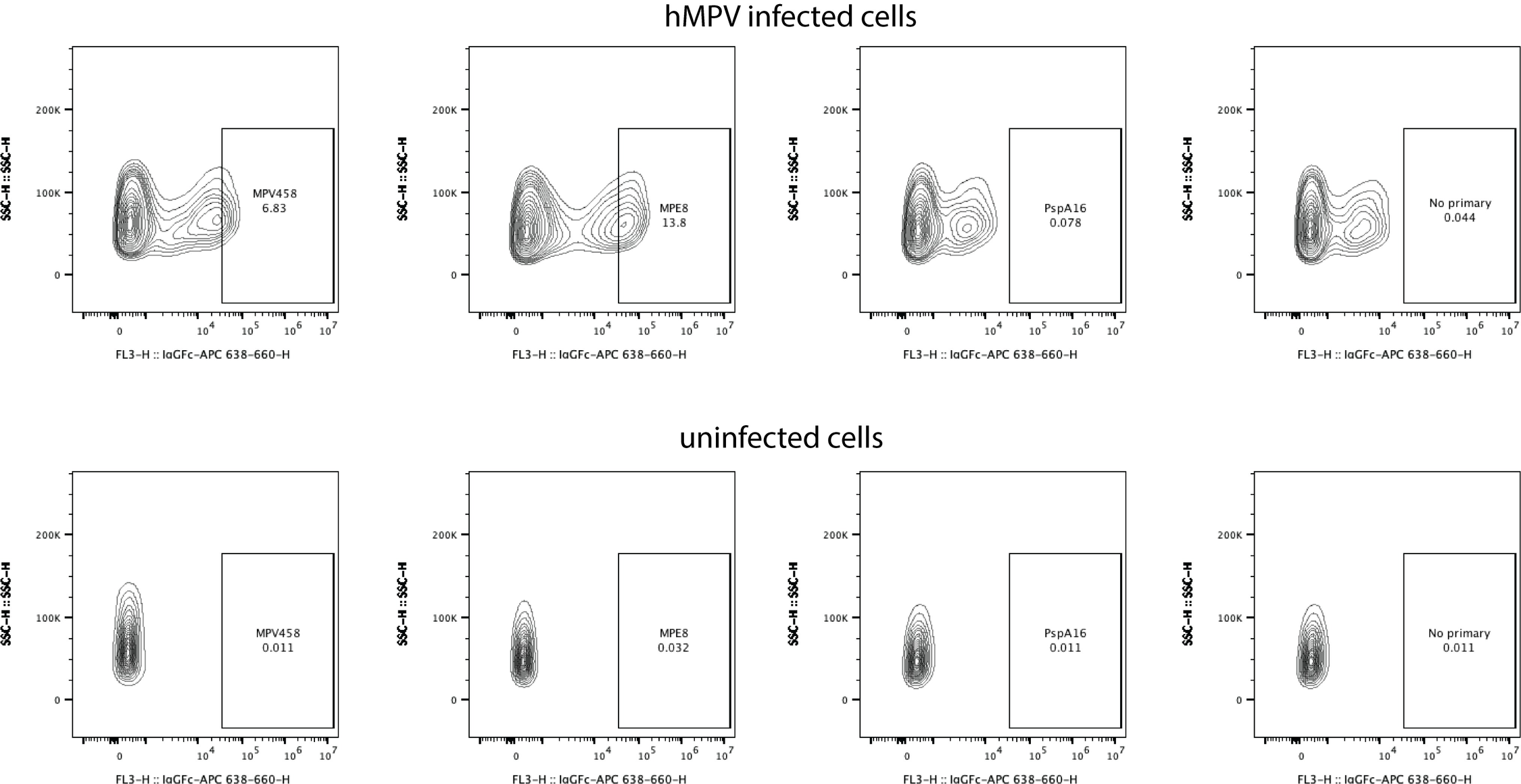

Supplement: S10 Fig — Twenty-four hours after infection, cells were harvested and stained with mAbs indicated. MPV458 and MPE8 induced a fluorescent shift in infected cells as compared to the pneumococcal-specific mAb PspA16. (TIF) [file ppat.1008942.s010.tif]
