# Supplementary material for: Antibody recognition of the Pneumovirus fusion protein trimer interface
Source: PLoS Pathog. 2020 Oct 9;16(10):e1008942. doi: 10.1371/journal.ppat.1008942 (PMC7598476; doi:10.1371/journal.ppat.1008942)
Supplement: S1 Table — (DOCX) [file ppat.1008942.s011.docx]

| **Table S1. Properties and sequences of recombinant hMPV F protein constructs used in this study.** | | | |
| --- | --- | --- | --- |
| **Construct name** | **Size**  **Major conformation** | **Trimerization domain** | **Sequence** |
| hMPV A1 F (NL/1/00) | Monomer | Foldon | MSWKVVIIFSLLITPQHGLKESYLEESCSTITEGYLSVLRTGWYTNVFTLEVGDVENLTCADGPSLIKTELDLTKSALRELRTVSADQLAREEQIENPRQS**KKRKRR**VATAAAVTAGVAIAKTIRLESEVTAIKNALKKTNEAVSTLGNGVRVLATAVRELKDFVSKNLTRAINKNKCDIADLKMAVSFSQFNRRFLNVVRQFSDNAGITPAISLDLMTDAELARAVSNMPTSAGQIKLMLENRAMVRRKGFGILIGVYGSSVIYMVQLPIFGVIDTPCWIVKAAPSCSEKKGNYACLLREDQGWYCQNAGSTVYYPNEKDCETRGDHVFCDTAAGINVAEQSKECNINISTTNYPCKVSTGRHPISMVALSPLGALVACYKGVSCSIGSNRVGIIKQLNKGCSYITNQDADTVTIDNTVYQLSKVEGEQHVIKGRPVSSSFDPVKFPEDQFNVALDQVFESIENSQALVDQSNRILSSAEKGNTSGRENLYFQGGGGGSGYIPEAPRDQAYVRKDGEWVLLSTFLGGTEGRHHHHHH |
| hMPV A2 F (NL/17/00) | Monomer | Foldon | MSWKVVIIFSLLITPQHGLKESYLEESCSTITEGYLSVLRTGWYTNVFTLEVGDVENLTCSDGPSLIKTELDLTKSALRELKTVSADQLAREEQIENPRQS**KKRKRR**VATAAAVTAGVAIAKTIRLESEVTAIKNALKTTNEAVSTLGNGVRVLATAVRELKDFVSKNLTRAINKNKCDIDDLKMAVSFSQFNRRFLNVVRQFSDNAGITPAISLDLMTDAELARAVSNMPTSAGQIKLMLENRAMVRRKGFGILIGVYGSSVIYTVQLPIFGVIDTPCWIVKAAPSCSEKKGNYACLLREDQGWYCQNAGSTVYYPNEKDCETRGDHVFCDTAAGINVAEQSKECNINISTTNYPCKVSTGRHPISMVALSPLGALVACYKGVSCSIGSNRVGIIKQLNKGCSYITNQDADTVTIDNTVYQLSKVEGEQHVIKGRPVSSSFDPIKFPEDQFNVALDQVFENIENSQALVDQSNRILSSAEKGNTSGRENLYFQGGGGGSGYIPEAPRDQAYVRKDGEWVLLSTFLGGTEGRHHHHHH |
| hMPV B1 F (NL/1/99) | Monomer | Foldon | MSWKVMIIISLLITPQHGLKESYLEESCSTITEGYLSVLRTGWYTNVFTLEVGDVENLTCTDGPSLIKTELDLTKSALRELKTVSADQLAREEQIENPRQS**KKRKRR**VATAAAVTAGIAIAKTIRLESEVNAIKGALKQTNEAVSTLGNGVRVLATAVRELKEFVSKNLTSAINRNKCDIADLKMAVSFSQFNRRFLNVVRQFSDNAGITPAISLDLMTDAELARAVSYMPTSAGQIKLMLENRAMVRRKGFGILIGVYGSSVIYMVQLPIFGVIDTPCWIIKAAPSCSEKNGNYACLLREDQGWYCKNAGSTVYYPNEKDCETRGDHVFCDTAAGINVAEQSRECNINISTTNYPCKVSTGRHPISMVALSPLGALVACYKGVSCSIGSNWVGIIKQLPKGCSYITNQDADTVTIDNTVYQLSKVEGEQHVIKGRPVSSSFDPIKFPEDQFNVALDQVFESIENSQALVDQSNKILNSAEKGNTSGRENLYFQGGGGGSGYIPEAPRDQAYVRKDGEWVLLSTFLGGTEGRHHHHHH |
| hMPV B2 F (NL/1/94) | Monomer | Foldon | MSWKVMIIISLLITPQHGLKESYLEESCSTITEGYLSVLRTGWYTNVFTLEVGDVENLTCTDGPSLIKTELDLTKSALRELKTVSADQLAREEQIENPRQS**KKRKRR**VATAAAVTAGIAIAKTIRLESEVNAIKGALKTTNEAVSTLGNGVRVLATAVRELKEFVSKNLTSAINKNKCDIADLKMAVSFSQFNRRFLNVVRQFSDNAGITPAISLDLMTDAELARAVSYMPTSAGQIKLMLENRAMVRRKGFGILIGVYGSSVIYMVQLPIFGVIDTPCWIIKAAPSCSEKDGNYACLLREDQGWYCKNAGSTVYYPNEKDCETRGDHVFCDTAAGINVAEQSRECNINISTTNYPCKVSTGRHPISMVALSPLGALVACYKGVSCSIGSNRVGIIKQLPKGCSYITNQDADTVTIDNTVYQLSKVEGEQHVIKGRPVSSSFDPIRFPEDQFNVALDQVFESIENSQALVDQSNKILNSAEKGNTSGRENLYFQGGGGGSGYIPEAPRDQAYVRKDGEWVLLSTFLGGTEGRHHHHHH |
| hMPV B2 F-GCN4 (TN/99-419) | Trimer, mixture of pre-fusion and post-fusion | GCN4 | MIIISLLITPQHGLKESYLEESCSTITEGYLSVLRTGWYTNVFTLEVGDVENLTCTDGPSLIKTELDLTKSALRELKTVSADQLAREEQIENP**RQSR**FVLGAIALGVATAAAVTAGIAIAKTIRLESEVNAIKGALKTTNEAVSTLGNGVRVLATAVRELKEFVSKNLTSAINKNKCDIADLKMAVSFSQFNRRFLNVVRQFSDNAGITPAISLDLMTDAELARAVSYMPTSAGQIKLMLENRAMVRRKGFGILIGVYGSSVIYMVQLPIFGVIDTPCWIIKAAPSCSEKDGNYACLLREDQGWYCKNAGSTVYYPNDKDCETRGDHVFCDTAAGINVAEQSRECNINISTTNYPCKVSTGRHPISMVALSPLGALVACYKGVSCSIGSNRVGIIKQLPKGCSYITNQDADTVTIDNTVYQLSKVEGEQHVIKGRPVSSSFDPIKFPEDQFNVALDQVFESIENSQALVDQSNKILNSAEARMKQIEDKIEEILSKIYHIENEIARIKKLIGEAGSGENLYFQGGSGGHHHHHHHH |
| hMPV 130-BV (NL/1/00) | Trimer, mixture of pre-fusion and post-fusion | Foldon | MSWKVVIIFSLLITPQHGLKESYLEESCSTITEGYLSVLRTGWYTNVFTLEVGDVENLTCADGPSLIKTELDLTKSALRELRTVSADQLAREEQIENP**RQSR**FVLGAIALGVATAAAVTAGVAIAKTIRLESEVTAIKNALKKTNEAVSTLGNGVRVLATAVRELKDFVSKNLTRAINKNKCDIPDLKMAVSFSQFNRRFLNVVRQFSDNAGITPAISLDLMTDAELARAVSNMPTSAGQIKLMLENRAMVRRKGFGILIGVYGSSVIYMVQLPIFGVIDTPCWIVKAAPSCSEKKGNYACLLREDQGWYCQNAGSTVYYPNEKDCETRGDHVFCDTAAGINVAEQSKECNINISTTNYPCKVSTGRHPISMVALSPLGALVACYKGVSCSIGSNRVGIIKQLNKGCSYITNQDADTVTIDNTVYQLSKVEGEQHVIKGRPVSSSFDPVKFPEDQFNVALDQVFESIENSQALVDQSNRILSSAESAIGGYIPEAPRDGQAYVRKDGEWVLLSTFLGGLVPRGSHHHHHH |
| The cleavage site in each construct is indicated in bold in the sequence.  The 66-87 sequence for each strain is labeled in red. | | | |
