# Supplementary material for: Antibody recognition of the Pneumovirus fusion protein trimer interface
Source: PLoS Pathog. 2020 Oct 9;16(10):e1008942. doi: 10.1371/journal.ppat.1008942 (PMC7598476; doi:10.1371/journal.ppat.1008942)
Supplement: S2 Table — (DOCX) [file ppat.1008942.s012.docx]

| **Table S2. IMGT V-QUEST Analysis of MPV458 and MPV465.** | | |
| --- | --- | --- |
| **Gene** | **MPV458 IgG3/kappa** | **MPV465 IgG1/lambda** |
| **V_H_**  **% identity** | IGHV3-30*03  IGHV3-30*18  IGHV3-30-5*01  89.6% | IGHV3-33*01  IGHV3-33*06  IGHV3-33*07  90.3% |
| **D_H_** | IGHD2-2*01 | IGHD3-22*01 |
| **J_H_**  **% identity** | IGHJ3*01  IGHJ3*02  87.8% | IGHJ5*02 F  90.2% |
| **V_L_** | IGKV1-33*01 IGKV1D-33*01  94.3% | IGLV1-47*02 F  95.6% |
| **J_L_** | IGKJ5*01 F  89.5% | IGLJ3*02  97.1% |
| **CDR-H1** | GFDFSRYG | GFTFGTYG |
| **CDR-H2** | IVYAGSNK | IWLDGSKT |
| **CDR-H3** | ARDQAFDL | ARAPGSVWYDTRGHMKGWFDP |
| **CDR-L1** | QGISRS | SSNIENNY |
| **CDR-L2** | DAS | GDN |
| **CDR-L3** | QQYDNLRIS | ATWDDNLSGPV |
