# Supplementary material for: Antibody recognition of the Pneumovirus fusion protein trimer interface
Source: PLoS Pathog. 2020 Oct 9;16(10):e1008942. doi: 10.1371/journal.ppat.1008942 (PMC7598476; doi:10.1371/journal.ppat.1008942)
Supplement: S3 Table — (DOCX) [file ppat.1008942.s013.docx]

| Table S3. Data collection and refinement statistics. | |
| --- | --- |
|  | hMPV B2 F + MPV458 Fab |
| Wavelength | 1.000 Å |
| Resolution range | 41.05 - 3.1 (3.211 - 3.1) |
| Space group | *P* 6_5_ |
| Unit cell | 128.5 128.5 188.4 90 90 120 |
| Total reflections | 671484 (67401) |
| Unique reflections | 31943 (3223) |
| Multiplicity | 21.0 (20.9) |
| Completeness (%) | 99.91 (100.00) |
| Mean I/sigma(I) | 17.62 (1.58) |
| Wilson B-factor | 107.39 |
| R-merge | 0.1407 (2.188) |
| R-meas | 0.1443 (2.243) |
| R-pim | 0.03155 (0.4889) |
| CC1/2 | 0.999 (0.621) |
| CC* | 1 (0.875) |
| Reflections used in refinement | 31931 (3223) |
| Reflections used for R-free | 3241 (350) |
| R-work | 0.1897 (0.3470) |
| R-free | 0.2339 (0.4018) |
| CC(work) | 0.960 (0.680) |
| CC(free) | 0.958 (0.511) |
| Number of non-hydrogen atoms | 6265 |
| macromolecules | 6190 |
| ligands | 75 |
| Protein residues | 818 |
| RMS(bonds) | 0.011 |
| RMS(angles) | 1.26 |
| Ramachandran favored (%) | 92.82 |
| Ramachandran allowed (%) | 6.93 |
| Ramachandran outliers (%) | 0.25 |
| Rotamer outliers (%) | 0.00 |
| Clashscore | 11.10 |
| Average B-factor | 100.80 |
| macromolecules | 100.36 |
| ligands | 137.75 |
| Statistics for the highest-resolution shell are shown in parentheses. | |
